# Spatial transcriptomics reveals molecular dysfunction associated with cortical Lewy pathology

**DOI:** 10.1038/s41467-024-47027-8

**Published:** 2024-03-26

**Authors:** Thomas M. Goralski, Lindsay Meyerdirk, Libby Breton, Laura Brasseur, Kevin Kurgat, Daniella DeWeerd, Lisa Turner, Katelyn Becker, Marie Adams, Daniel J. Newhouse, Michael X. Henderson

**Affiliations:** 1 Department of Neurodegenerative Science, Van Andel Institute, Grand Rapids, MI 49503 USA; 2 Aligning Science Across Parkinson’s (ASAP) Collaborative Research Network, Chevy Chase, MD USA; 3 Van Andel Institute Pathology Core, Grand Rapids, MI 49503 USA; 4 Van Andel Institute Genomics Core, Grand Rapids, MI 49503 USA; 5 NanoString Technologies, Seattle, WA USA

**Keywords:** Molecular neuroscience, Parkinson's disease

## Abstract

A key hallmark of Parkinson’s disease (PD) is Lewy pathology. Composed of α-synuclein, Lewy pathology is found both in dopaminergic neurons that modulate motor function, and cortical regions that control cognitive function. Recent work has established the molecular identity of dopaminergic neurons susceptible to death, but little is known about cortical neurons susceptible to Lewy pathology or molecular changes induced by aggregates. In the current study, we use spatial transcriptomics to capture whole transcriptome signatures from cortical neurons with α-synuclein pathology compared to neurons without pathology. We find, both in PD and related PD dementia, dementia with Lewy bodies and in the pre-formed fibril α-synucleinopathy mouse model, that specific classes of excitatory neurons are vulnerable to developing Lewy pathology. Further, we identify conserved gene expression changes in aggregate-bearing neurons that we designate the Lewy-associated molecular dysfunction from aggregates (LAMDA) signature. Neurons with aggregates downregulate synaptic, mitochondrial, ubiquitin-proteasome, endo-lysosomal, and cytoskeletal genes and upregulate DNA repair and complement/cytokine genes. Our results identify neurons vulnerable to Lewy pathology in the PD cortex and describe a conserved signature of molecular dysfunction in both mice and humans.

## Abstract (web summary)

The impact of α-synuclein aggregates on neurons has been unclear. Here, the authors identify a Lewy Associated Molecular Dysfunction from Aggregates (LAMDA) signature in inclusion bearing neurons in human brain and a mouse model of α-synucleinopathy.

## Introduction

Parkinson’s disease (PD) is diagnosed post-mortem by loss of dopaminergic neurons in the substantia nigra pars compacta (SNc) and the presence of α-synuclein Lewy pathology throughout the brain^1^. Yet, it is unclear what happens in neurons with Lewy bodies (LBs), and some have argued that these large cytoplasmic inclusions may even be protective (reviewed here^2^). Further, outside of the SNc, there is minimal knowledge about which neurons are susceptible to developing Lewy pathology. The current study aimed to discover which neurons bear α-synuclein inclusions and what molecular processes are changed in these neurons. To accomplish this, we used spatial transcriptomics and complementary approaches in PD brain and a mouse model of α-synucleinopathy.

PD is diagnosed clinically by the presence of motor dysfunction^1^, however, PD patients experience many other symptoms. One of the most devastating symptoms of PD is the progression of over 80 percent of patients to dementia^3^, a diagnosis associated most strongly with progression of Lewy pathology to cortical regions^4–8^. Many brainstem nuclei sustain neuron loss in PD, and vulnerability factors have been identified that unite several of these nuclei—autonomous pacemaking activity, hyperbranching axons, and synthesis of catecholaminergic neurotransmitters that may promote mitochondrial dysfunction^9,10^. Yet, even within these highly susceptible types of neurons, there is a selective vulnerability. Early morphological studies found that the ventral tier of the SNc is particularly vulnerable to neuron loss in PD^11,12^. Advances in RNA sequencing have shown that *SOX6/AGTR1*-positive neurons are selectively lost in the SNc^13^ and there is a dysfunctional transcriptomic signature associated with SNc neurons in PD^13–15^. Yet, these single-cell RNA sequencing (scRNAseq) studies did not assess molecular processes in neurons bearing Lewy pathology.

Staging studies have shown that Lewy pathology extends past the brainstem to neocortical regions in later stages, especially cingulate and prefrontal cortex^16^. Within those regions, there is a sparse distribution of affected neurons mostly in deep layers^16,17^, suggesting that subpopulations of neurons in those layers are particularly affected. These appear to be cortico-cortical projection neurons because both short-axoned projection neurons, inhibitory interneurons, and large myelinated cortico-spinal neurons do not bear most Lewy pathology^18,19^. This has suggested that, as with catecholaminergic neurons, a long axon is a potential source of vulnerability, and myelination of corticospinal neurons may be protective^18^.

We hypothesized that there are select populations of neurons vulnerable to developing Lewy pathology in PD and that those neurons would show disruption of cellular processes preceding their loss. To separate α-synuclein inclusion-bearing from resilient neurons, we used immunostaining and spatial transcriptomics to collect whole-transcriptome molecular signatures from those two populations in PD brains and the pre-formed fibril (PFF) mouse model of α-synucleinopathy. We were able to clearly identify transcriptomic changes by cortical layer and by inclusion status. Layer 5 intratelencephalic (IT) and layer 6b neurons were particularly vulnerable, while inhibitory and pyramidal tract (PT) neurons were resilient. Further, inclusion-bearing neurons had reduced synaptic, mitochondrial, proteasomal, endo-lysosomal, and cytoskeletal gene expression, while genes associated with DNA damage repair, apoptosis, and complement cascade were elevated. Molecular changes in LB-bearing neurons showed remarkable similarity to those seen in the α-synucleinopathy mouse model, with over 600 conserved gene expression changes. Together, this study indicates that there is a selectively vulnerable population of neurons in PD cortex, and α-synuclein inclusions are associated with a strong and conserved stress response in neurons. Future studies may take advantage of this knowledge to target specific classes of neurons and molecular pathways for therapeutic effect.

## Results

### Collection of whole transcriptomes of aggregate-bearing neurons in Lewy body disease

Single-cell transcriptomics has enabled the examination of gene signatures associated with PD^13–15,20,21^. However, none of these studies have examined whether the isolated neurons bear α-synuclein inclusions. Isolating inclusions is difficult using such techniques due to the necessary dissociation of cells and loss of cytoplasm in most post-mortem human brain studies. The preponderance of evidence suggests that neurons with LBs undergo cellular dysfunction and cell death, but some researchers have argued that inclusions may be protective (reviewed^2^), sequestering toxic oligomeric species. We therefore employed a recently-developed Whole Transcriptome Atlas capture based on in situ hybridization technology using the GeoMx digital spatial profiler (DSP)^22^.

**Fig. 1:**
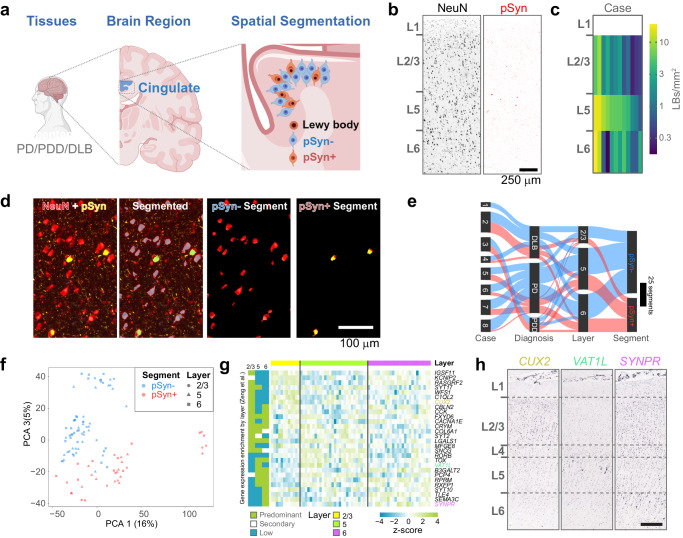
Collection of transcriptome signatures of neurons with or without Lewy pathology from human cingulate cortex. **a** Experimental schematic. Human cingulate cortex tissue was collected from 8 individuals diagnosed with idiopathic PD, PDD, or DLB. Created with BioRender.com. **b** Example NeuN and pSyn staining from cingulate cortex. Laminar distribution of Lewy pathology can vary between patients with L5 showing the most prominent pathology across cases. (n = 8 cases) Scale bar = 250 μm. **c** Quantification of the average number of Lewy bodies per area cingulate cortex used in this study. Layer 5 showed the highest degree of pathology. **d** A representative brain section stained for NeuN (red), GFAP (cyan), and pSyn (yellow) with a demonstration of the segmentation strategy (n = 8 cases). Scale bar = 100 μm. **e** Sankey plot of all areas of interest segments that passed quality control. **f** Principal component analysis plot demonstrating the ability of PCA 1 and 2 to largely segregate pSyn- and pSyn+ segments, with different cortical layers clustering together. **g** Gene expression heatmap for genes significantly differentially expressed by cortical layer in the human GeoMx dataset. Layer specific gene enrichment is noted on the left from Zeng et al.^26^. **h** In situ hybridization images of three genes differentially enriched in layer 2/3 (*CUX2*), layer 5 (*VAT1L*) or layer 6 (*SYNPR*) of human dorsolateral prefrontal cortex. Images adapted from the Allen Brain Atlas in situ data (https://human.brain-map.org/ish/specimen/show/80935564). Scale bar = 500 µm. Source data are provided as a Source Data file.

We examined neurons with and without LBs in human disease brain (Fig. 1a). The cingulate gyrus is one of the first cortical structures to develop Lewy pathology in PD, and one of the most severely affected^23^. We identified 25 cases with a neuropathological diagnosis of PD, Parkinson’s disease dementia (PDD), or dementia with Lewy bodies (DLB) that were likely to have high cortical LBs and low tau pathology for inclusion in our study. All but two cases were classified as unified Lewy body stage IV (neocortical)^24^ with two cases stage III (brainstem/limbic). In addition, all cases were tau Braak stage IV or below^25^. We stained all 25 brains for pS129 α-synuclein, pS202/T205 tau, pS409/410 TDP-43, and amyloid β. Of the 25, 16 brains either had minimal Lewy bodies in the cingulate cortex, or also had tau tangles (Supplementary Fig. 1). These cases were considered unviable for spatial transcriptomics because neurons with tau tangles could be errantly classified as unaffected with pathology. The remaining 9 cases showed abundant Lewy pathology, primarily in layer 5, with less pathology in layers 6 and 2/3 (Figs. 1b, c). Three additional cases had Lewy bodies too sparse for spatial transcriptomics but were included in subsequent validation experiments.

To segment neurons with and without pathology, sections were stained with neuron marker NeuN, astrocyte marker glial fibrillary acidic protein (GFAP), and pathology marker pS129 α-synuclein (pSyn) and imaged on the GeoMx DSP. While NeuN is reported as a marker of neuronal nuclei in human and mouse tissue, it also labels neuronal cell bodies (Supplementary Fig. 2). ROIs were manually drawn within specific cortical layers, and neurons with and without LBs were segmented using NeuN and pSyn fluorescence. Two populations were collected: NeuN + /pSyn- (referred to as **pSyn-**) or NeuN + /pSyn+ (referred to as **pSyn** + , Fig. 1d). Segments were filtered, and segments that did not meet the following criteria were removed: for sequence stitching <80%, aligned sequence reads <75%, sequence saturation <50%, and <5% of genes being detected above the LOQ. Of the 18815 gene targets contained in the GeoMx Human Whole Transcriptome Atlas, 8601 were included in the downstream analyses following QC analysis. Genes were removed based on a global outlier (Grubbs test, p < 0.01), local outliers (Grubbs test, p < 0.01), limit of quantification assessment (Supplementary Fig. 3). Together, we were able to collect transcript signatures from 8 brains that passed quality control (Fig. 1e). Brains were from patients at an average of 78 years old and with an average post-mortem interval of 3.9 hours (Supplementary Fig. 4). Six were male, two were female. pSyn- and pSyn+ segments were the main driver of heterogeneity in these samples, and a principal component analysis was able to mostly separate the two segments (Fig. 1f).

We sought to assess the ability to distinguish cortical layer-selective gene signatures from the pSyn- segment. While these signatures are not as well established as they are within the mouse cortex, we used the work of Zeng and colleagues to characterize layer-selective gene expression in human cortex^26^ (Fig. 1g). We cross-referenced their layer-selective genes with genes in our dataset that showed significant gene expression differences by cortical layer. We identified 28 genes present in both datasets, and the expression patterns were largely replicated (Fig. 1g). We evaluated the expression pattern of three of the differentially-expressed genes (DEGs) by examining in situ hybridization patterns from the dorsolateral prefrontal cortex of Allen Institute cohorts. Cingulate cortex, which does not have a prominent layer 4, was not available. We could see the enrichment of *CUX2* in layers 2/3, *VAT1L* in layer 5, and *SYNPR* in layer 6 (Fig. 1h), in agreement with GeoMx expression patterns. These results demonstrate the ability of this method to collect high quality, spatially resolved transcripts in PD brain.

### Aggregate-bearing neurons in Lewy body disease show gene expression changes associated with cellular dysfunction

**Fig. 2:**
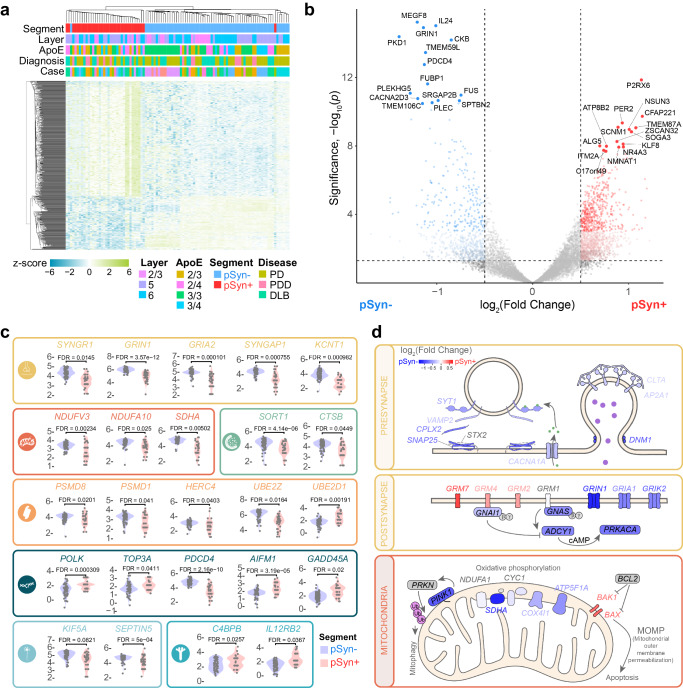
LB-bearing neurons show gene expression changes associated with cellular dysfunction. **a** A heatmap displaying the z-score of all genes differentially expressed between pSyn- and pSyn+ segments with false discovery rate (FDR)-corrected p-value < 0.01. Both rows and columns have been sorted by unsupervised clustering. **b** Volcano plot comparing genes differentially expressed enriched in pSyn- segments (blue) and pSyn+ segments (red) across regions as determined by two tailed mixed effects linear regression with multiple test correction using the FDR. **c** Examples of 25 genes that are differentially expressed in pSyn- and pSyn+ segments that fit into one of 9 categories as noted by the color and corresponding symbol. From top-to-bottom, left-to-right, categories are synapse, mitochondria, lysosome, ubiquitin-proteasome, DNA damage-apoptosis, cytoskeleton, complement-cytokine. **d** Example pathways disrupted in LB-bearing neurons with relative gene expression plotted by color for each gene in the 3 pathways.

We next investigated gene expression changes associated with LBs. We conducted DEG analysis in pSyn- versus pSyn+ segments and identified 1422 genes that were upregulated in pSyn segments and 620 genes that were downregulated in pSyn segments after false discovery rate correction. Hierarchical clustering of segments was able to clearly separate pSyn- and pSyn+ segments by gene expression patterns (Fig. 2a). Some of the top genes upregulated in pSyn neurons were related to complement/cytokine (*C4BPB, IL12RB2*), DNA/RNA integrity (*POLK, TOP3A, AIFM1, GADD45A, NSUN3*), and ion channels (*P2RX6, SCNM1*), among others (Fig. 2b, c). Top genes downregulated in pSyn neurons were related to the synapse (*SYNGR1, GRIN1, GRIA2, SYNGAP1, KCNT1*), mitochondria (*NDUFV3, NDUFA10, SDHA*), metabolism (*CKB*), membrane signaling (*CACNA2D3, TMEM59L, TMEM106C*), endo-lysosome (*SORT1, CTSB*), ubiquitin-proteasome (*PSMD8, PSMD1, HERC4, UBE2Z*), and cytoskeleton (*SPTBN2, KIF5A, SEPTIN5, PLEC*) among others (Fig. 2b, c).

To determine if differentially expressed genes were enriched in particular pathways, we performed gene set enrichment analysis using KEGG Brite pathways^27–29^ comparing pSyn+ and pSyn- segments (Fig. 2d, Supplementary Fig. 5). Pathways downregulated in LB-bearing neurons related to the synapse (endocytosis, retrograde endocannabinoid signaling), metabolism (sphingolipid signaling pathway), cell signaling (Ca^2+^/calmodulin-dependent kinase, Wnt signaling pathway), RNA (mRNA surveillance pathway, spliceosome, RNA binding proteins, hnRNP proteins, ribosome, small subunit), and neurodegenerative disease (Parkinson’s). Pathways upregulated in LB-bearing neurons related to complement cascade/cytokine (class II cytokine receptor, cytokine-cytokine receptor interaction, complement and coagulation cascades), DNA integrity/apoptosis (base excision repair, mismatch repair), and metabolism (steroid biosynthesis, n-glycan biosynthesis, histidine metabolism, ascorbate and aldarate, tryptophan). Together, differential gene expression between pSyn- and pSyn+ neurons revealed that there is broad cellular dysfunction in neurons bearing inclusions with downregulation of pathways essential for neuronal function, and upregulation of stress-related pathways. However, these brains represent end-stage disease, making it difficult to know how long neurons have had inclusions and understand how LB formation may impact neurons over short time scales. Therefore, we turned to a mouse model of α-synucleinopathy to determine if the observed transcription changes were broadly applicable and to enable more in-depth mechanistic analysis.

### Collection of whole transcriptome signatures of neurons with or without pathology from mouse cortex

LB-like pathology can be induced in wildtype mice via injection of α-synuclein pre-formed fibrils (PFFs) into the brain^30,31^. These mice do not require the overexpression or mutation of any protein. Instead, injection of misfolded α-synuclein is internalized by neurons and induces the formation of Lewy pathology-like inclusions in wildtype mice^30^ or primary neurons^32^. These inclusions contain endogenous fibrillar α-synuclein phosphorylated at S129. They are also decorated with ubiquitin and p62 and are proteinase-resistant and detergent-insoluble. These inclusions undergo maturation^33,34^, eventually accumulating lipid membranes and organelles, similar to Lewy bodies^34^, and lead to neuron death^32–35^. In most ways that have been measured, these LB-like inclusions resemble human LBs, however, the mouse model provides additional spatiotemporal control unavailable in humans. By defining the injection site and the time post-injection, exact timepoints in progression can be sampled. We have previously demonstrated that dorsal striatum injection of α-synuclein PFFs leads to a broad distribution of pathology throughout the mouse brain that is constrained by anatomical connectivity and regional vulnerability^31^. Importantly for this study, there is substantial pathology in this model in frontal cortical regions.

**Fig. 3:**
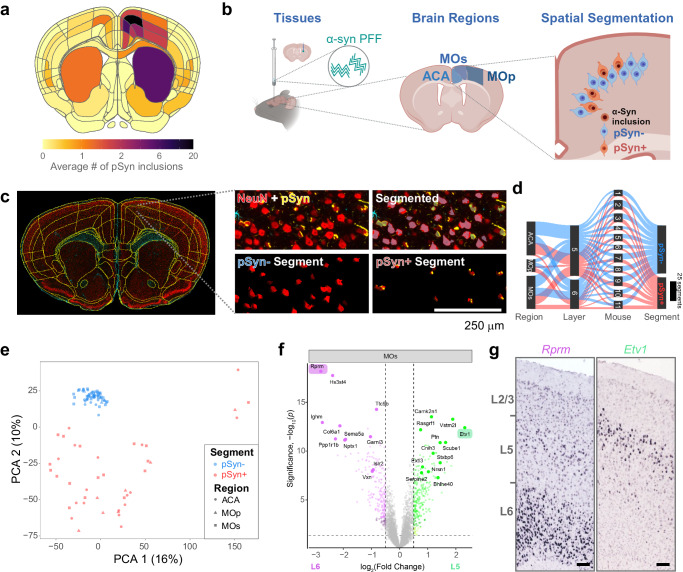
Collection of transcriptome signatures of neurons with or without pathology from mouse cortex. **a** Quantification of the average number of pSyn inclusions per region from 12 mice injected with α-synuclein PFFs. ACA, MOs, and MOp had the highest pathology burden, especially cortical layer 5. **b** Experimental schematic. Mice were injected in the dorsal caudoputamen with α-synuclein PFFs. After 3 months, the brains of mice were removed and brain sections were prepared for spatial transcriptomic analysis, with a focus on frontal cortical regions—anterior cingulate (ACA), secondary motor (MOs), and primary motor (MOp). Within each region, pSyn- or pSyn+ neurons were identified by the presence or absence of α-synuclein pathology and RNA from those regions were selectively identified. Created with BioRender.com. **c** A representative brain section stained for NeuN (red), GFAP (cyan), and pSyn (yellow) registered to the Allen Brain Atlas CCFv3 (10.1016/j.cell.2020.04.007). A zoomed view of MOs/ACA is shown denoting layers of the cortex and a demonstration of the segmentation strategy is viewed at far right. Scale bar = 250 μm. **d** Sankey plot of all areas of interest segments that passed quality control (*n* = 11 mice). **e** Principal component analysis plot demonstrating the ability of PCA 1 and 2 to segregate pSyn- and pSyn+ segments, with different regional segments clustering together. **f** Volcano plot comparing genes differentially expressed between pSyn- segments in layer 5 (green) and layer 6 (magenta) of MOs identifies genes know to be differentially expressed in the different cortical layers as determined by two tailed mixed effects linear regression with multiple test correction using FDR. Two such genes are highlighted. **g** In situ hybridization images of two genes differentially enriched in layer 6 (*Rprm*) or layer 5 (*Etv1*) of mouse MOs. Images adapted from the Allen Brain Atlas in situ data (*Rprm*: https://mouse.brain-map.org/gene/show/43717, *Etv1*: https://mouse.brain-map.org/gene/show/13786).

We first sought to characterize the cortical pathology more deeply in α-synuclein PFF-injected mice to identify a region suitable for spatial transcriptomics analysis. Mice were aged 3 months post-injection and rostral brain regions were stained for pS129 α-synuclein, and the number of inclusions was quantified using an adapted version of the QUINT workflow^36,37^. Mice developed a reproducible distribution of α-synuclein inclusions in frontal cortical regions—anterior cingulate (ACA), secondary motor (MOs), and primary motor (MOp, Fig. 3a) cortex. Further, inclusion burden was most densely distributed in cortical layer 5 of these regions. We therefore proceeded to perform GeoMx DSP whole transcriptome analysis in these three regions (Fig. 3b), segmenting pSyn+ and pSyn- neurons. We used the same three morphology markers as used in human cingulate cortex: NeuN, GFAP, and pS129 α-synuclein (Fig. 3c, Supplementary Fig. 6). To accurately annotate cortical layers, brain section images were downloaded from the GeoMx instrument, registered to the Allen Brain Atlas CCFv3 using a modified version of the QUINT workflow^36,37^, and registrations were overlaid on the slide image in the GeoMx instrument (Fig. 3c). Regions of interest were drawn within Allen Brain Atlas regions. Segmentations were developed to identify two classes of neurons, those that were NeuN+/pSyn- and those that were NeuN+/pSyn+ (Fig. 3c). These two classes are referred to as pSyn- (pathology free) and pSyn+ (pathology-bearing) for the remainder of the manuscript.

Using this methodology, we collected 103 segments that passed quality control from 11 mice (Fig. 3d). Most segments were collected from the ACA and MOs, with MOp having fewer neuronal inclusions, and therefore fewer high-quality segments. Segments were filtered, and segments that did not meet the following criteria were removed: for sequence stitching <80%, aligned sequence reads <75%, sequence saturation <50%, and <5% of genes being detected above the limit of quantification (LOQ). Of the 20175 gene targets contained in the GeoMx Mouse Whole Transcriptome Atlas, 9035 were included in the downstream analyses following QC analysis. Genes were removed based on a global outlier (Grubbs test, *p* < 0.01), local outliers (Grubbs test, *p* < 0.01), and limit of quantification assessment (Supplementary Fig. 7). The pSyn- and pSyn+ segments were the major drivers in a principal component analysis (Fig. 3E), with cortical layer and region showing less separation.

To validate the ability of the captured transcriptome data to differentiate neuron types, we first assessed differentially expressed genes (DEGs) between layers 5 and 6 in the pSyn- segment. We identified many DEGs in MOs (Fig. 3f), ACA (Supplementary Fig. 8a), and MOp (Supplementary Fig. 8b). Many of the DEGs were previously identified to be layer-enriched, including two of the most highly DEGs, *Rprm* (enriched in layer 6) and *Etv1* (enriched in layer 5). Enrichment of gene expression was confirmed by assessing in situ hybridization staining patterns established by the Allen Institute^38^ (Fig. 3g).

### α-Synuclein inclusion-bearing neurons show gene expression changes associated with cellular dysfunction

**Fig. 4:**
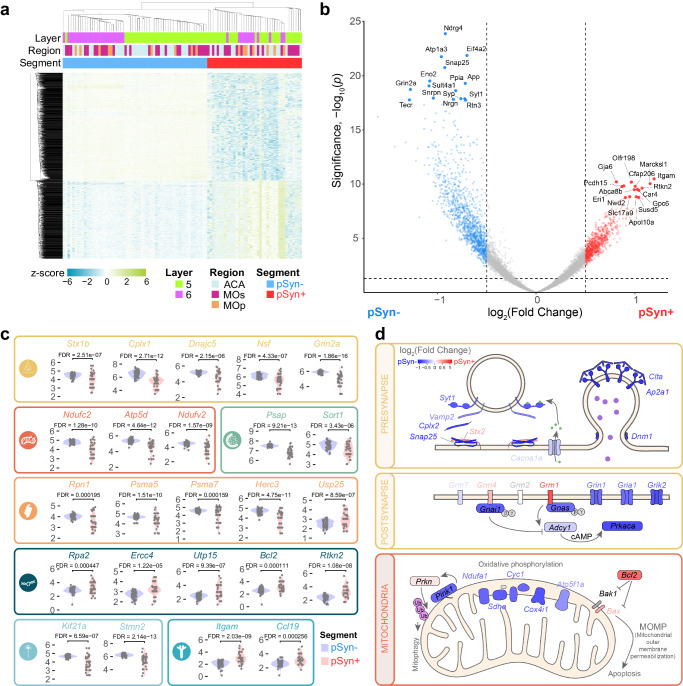
α-Synuclein inclusion-bearing neurons show gene expression changes associated with cellular dysfunction. **a** A heatmap displaying the z-score of all genes differentially expressed between pSyn- and pSyn+ segments with FDR-corrected *p*-value < 0.01. Both rows and columns have been sorted by unsupervised clustering. (*n* = 11 mice) **b** Volcano plot comparing genes differentially expressed enriched in pSyn- segments (blue) and pSyn+ segments (red) across regions as determined by two tailed mixed effects linear regression with multiple test correction using the FDR. **c** Examples of 25 genes that are differentially expressed in pSyn- and pSyn+ segments that fit into one of 9 categories as noted by the color and corresponding symbol. From top-to-bottom, left-to-right, categories are synapse, mitochondria, lysosome, ubiquitin-proteasome, DNA damage-apoptosis, cytoskeleton, complement-cytokine. **d** Example pathways that are disrupted in LB-bearing neurons with relative gene expression plotted by color (blue: enriched in pSyn-, red: enriched in pSyn+) for each gene in the 3 pathways.

We next sought to identify gene expression changes associated with pSyn inclusions. We conducted DEG analysis in pSyn- versus pSyn+ segments and identified 2298 genes that were upregulated in pSyn+ segments and 2412 genes that were downregulated in pSyn+ segments after false discovery rate correction. Hierarchical clustering of segments was able to clearly separate pSyn- and pSyn+ segments by gene expression patterns (Fig. 4a). Some of the top genes upregulated in pSyn neurons were related to complement/cytokine (*Itgam*, *Ccl19*), DNA/RNA integrity (*Rpa2, Ercc4, Utp15, Bcl2, Rtkn2*), among others (Figs. 4b, c). Top genes downregulated in pSyn neurons were related to the synapse (*Stx1b*, *Cplx1, Dnajc5*, *Nsf*, *Grin2a, Snap25, App, Syt1, Syp*), mitochondria (*Ndufc2, Atp5d, Ndufv2*), lipid metabolism (*Tecr*), endo-lysosome (*Atp6ap2, Psap, Sort1*), ubiquitin-proteasome (*Rpn1, Psma5, Psma7, Herc3*) and cytoskeleton (*Nefm, Brsk1, Kcnc1, Septin7, Kif21a, Stmn2*) among others (Fig. 4b, c). Differential expression also appeared highly reproducible across regions (Supplementary Fig. 9).

To determine if differentially expressed genes were enriched in specific pathways, we performed gene set enrichment analysis using KEGG Brite pathways^27–29^ comparing pSyn+ and pSyn- segments (Fig. 4D, Supplementary Fig. 10). Pathways downregulated in inclusion-bearing neurons related to the synapse (glutamatergic synapse, dopaminergic synapse, GABAergic synapse, retrograde endocannabinoid signaling, endocytosis), mitochondria (NADH dehydrogenase, NADH:ubiquinone reductase, oxidative phosphorylation), metabolism (citrate cycle), cell signaling (phosphatidylinositol signaling system), RNA (eIF-3, RNA transport), ubiquitin-proteasome (proteasome, family T1 proteasome family, ubiquitin mediated proteolysis), and neurodegenerative disease (Parkinson’s, Alzheimer’s, Huntington’s). Pathways upregulated in inclusion-bearing neurons related to complement cascade/cytokine (CD molecules, class I cytokine receptor, cytokine-cytokine receptor interaction, complement and coagulation cascades), DNA integrity/apoptosis (apoptosis, small cell lung cancer, Fanconi anemia pathway, helicases), RNA (elongation associated factors), and metabolism (ascorbate and alderate, histidine, fatty acid degradation, hexosyltransferases, cysteine endopeptidases). Together, differential gene expression between pSyn- and pSyn+ neurons revealed that there is broad cellular dysfunction in neurons bearing inclusions with downregulation of pathways essential for neuronal function, and upregulation of stress-related pathways.

### Parkinson’s disease brain and mice have conserved signatures of α-synuclein inclusions

The α-synuclein PFF model and human tissue each have benefits and weaknesses. The mouse model is highly reproducible on a conserved genetic background, but neurons develop inclusions partially based on injection location. Human brain represents disease, but individuals have heterogenous genetic backgrounds and experience different environmental insults. To develop a clearer understanding of Lewy pathology-related changes, we investigated pathways and gene expression changes that were conserved in mice and humans. 321 and 293 conserved genes were down- and upregulated, respectively, in inclusion-bearing neurons in mice and humans (Supplementary Fig. 11). Due to the conservation of gene expression across brain regions in a mouse model and human Lewy body diseases, we consider this to be a Lewy-associated molecular dysfunction from aggregates (LAMDA) signature (Supplementary Fig. 11).

**Fig. 5:**
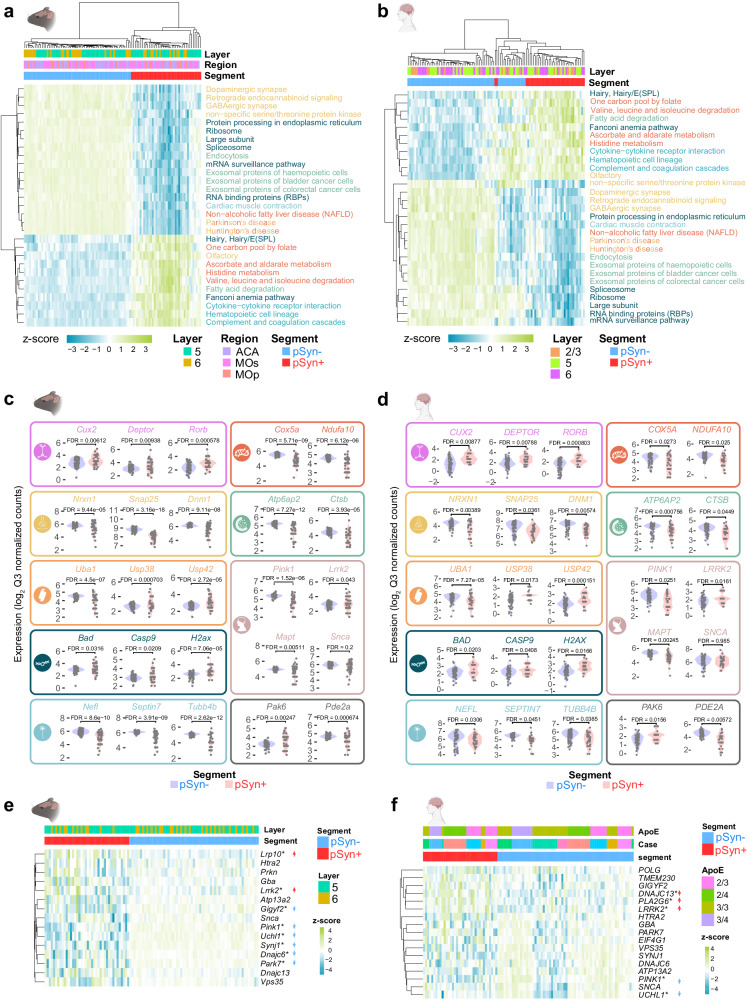
Parkinson’s disease brain and mice have conserved signatures of α-synuclein inclusions. **a** Gene set enrichment analysis was performed on pSyn- and pSyn+ segments in from α-synuclein PFF-injected mice. Z-scores of individual segments are plotted for each pathway. Only pathways conserved with human tissue are plotted here. (*N* = 11 mice). **b** Gene set enrichment analysis was performed on pSyn- and pSyn+ segments in from PD/PDD/DLB cingulate cortex. Z-scores of individual segments are plotted for each pathway. Only pathways conserved with mouse tissue are plotted here. (*n* = 8 cases)(**c**) Representative gene expression from several of the gene sets enriched in mouse pSyn- or pSyn+ segments are plotted. Categories included are (from top to bottom, left to right) cell type, mitochondria, synapse, lysosome, ubiquitin-proteasome, Parkinson’s disease, DNA integrity/apoptosis, cytoskeleton, other. **d** Representative gene expression from several of the gene sets enriched in human pSyn- or pSyn+ segments are plotted. Categories included are (from top to bottom, left to right) cell type, mitochondria, synapse, lysosome, ubiquitin-proteasome, Parkinson’s disease, DNA integrity/apoptosis, cytoskeleton, other. **e** Expression of genes related to monogenic PD in mice. *FDR-corrected *p*-value < 0.05 as determined by two tailed mixed effects linear regression with multiple test correction using FDR. Arrows indicate the direction of significant change. **f** Expression of genes related to monogenic PD in human cingulate. *FDR-corrected *p*-value < 0.05 as determined by two tailed mixed effects linear regression with multiple test correction using FDR. Arrows indicate the direction of significant change.

We identified 29 gene pathways that were significantly enriched in both mouse (Fig. 5a) and human (Fig. 5b) brain. Pathways downregulated in LAMDA related to the synapse, mitochondria, ubiquitin-proteasome, endo-lysosome, and cytoskeleton, while pathways upregulated in LAMDA related to DNA/RNA integrity and complement/cytokine. Conserved genes were identified that represent each of these pathways (Fig. 5c, d). In addition, we identified conserved cell type markers (*CUX2, DEPTOR, RORB*), PD-related genes (*PINK1, LRRK2, MAPT*), and cell signaling genes (*PAK6, PDE2A*, Fig. 5c, d).

We further explored the expression of genes associated with monogenic forms of PD^39^. In mice, two genes were elevated in inclusion-bearing neurons (*Lrp10, LRRK2*), while six genes were reduced (*Gigyf2, Pink1, Uchl1, Synj1, Dnajc6, Park7*, Fig. 5e). There was a general correspondence in the directionality of these changes in human cingulate, but with more heterogenous results (Fig. 5f). Expression of two genes (*PINK1, UCHL1*) was reduced, while expression of three genes (*DNAJC13, PLA2G6, LRRK2*) was increased. Together, these results indicate that there are conserved gene expression changes in mouse and human neurons bearing α-synuclein inclusions.

### α-Synuclein pathology is enriched in layer 5 intratelencephalic and layer 6b neurons

In addition to molecular changes in LB-bearing neurons, we were interested to see if we could determine the neuron types bearing inclusions. We first examined this question in human brain. Since we had collected multiple neurons per region, we performed cell deconvolution using the SpatialDecon^40^ algorithm to determine cell type proportion using the M1 − 10X Genomics dataset publicly available from the Allen Brain Institute^41^. The two main neuron types identified were VIP (inhibitory population) and L4 IT (excitatory population). Neurons with inclusions were estimated to have a lower abundance of VIP neurons, and a higher abundance of L4 IT neurons (Supplementary Fig. 12). This indicates that inhibitory neurons are less likely to bear inclusions, and excitatory (possibly intratelencephalic (IT) neurons) are more likely to bear inclusions. Due to heterogeneity of human samples, several neuron types, including pyramidal tract (PT/ET) neurons, were not identified, and some populations were too low to estimate properly. We therefore extended our examination of impacted cell types to mouse brain.

**Fig. 6:**
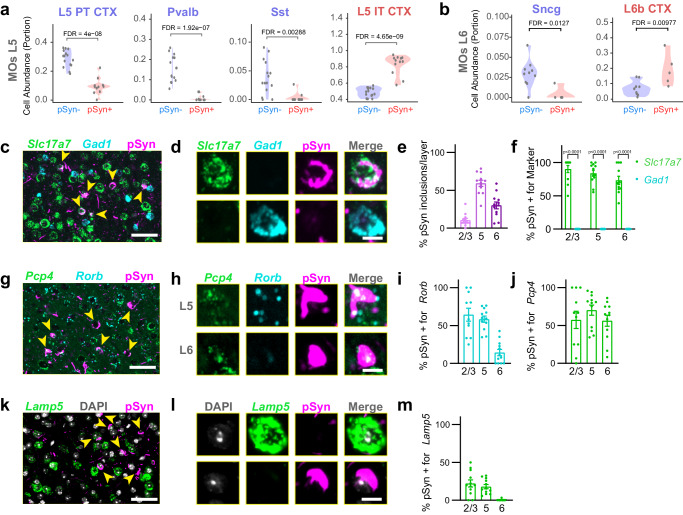
α-Synuclein pathology is enriched in layer 5 intratelencephalic and layer 6b neurons. **a** Cell abundance estimates from cell deconvolution analysis in layer 5 of MOs show enrichment of L5 PT, Pvalb, and Sst neurons in pSyn- segments and enrichment of L5 IT neurons in the pSyn+ neurons (*n* = 11 mice). **b** Cell abundance estimates from cell deconvolution analysis in layer 6 of MOs (*n* = 11 mice). show enrichment of Sncg cell types in pSyn- neurons and an enrichment of L6b neurons in pSyn+ neurons. Each dot is an individual segment and FDR-corrected p-values are displayed for statistical comparison. **c** A zoomed in view of layer 5 of MOs with yellow arrowheads denoting neurons with pSyn inclusions. Scale bar = 50 µm. **d** Examples of single cells positive for *Slc17a7*, *Gad1*, and/or pSyn. Scale bar = 10 µm. **e** Percentage of pSyn inclusions in cortical layers as a percentage of the total inclusions. **f** Percentage of cells with pSyn inclusions also positive for either *Slc17a7* or *Gad1* in each cortical layer. *p*-values are displayed from a mixed-effects analysis with Sidak’s multiple comparisons test to compare *Slc17a7* and *Gad1* positive neurons. **g** A zoomed in view of layer 5 of MOs with yellow arrowheads denoting neurons with pSyn inclusions. Scale bar = 50 µm. **h** Examples of single cells positive for *Pcp4*, *Rorb*, and/or pSyn. Scale bar = 10 µm. **i** Percentage of cells with pSyn inclusions also positive for *Rorb* in each cortical layer. **j** Quantification of the percentage of cells with pSyn inclusions also positive for *Pcp4* in each cortical layer. **k** A zoomed in view of layer 5 of MOs with yellow arrowheads denoting neurons with pSyn inclusions. Scale bar = 50 µm. **l** Examples of single cells positive for *Lamp5* and/or pSyn. Scale bar = 10 µm. **m** Percentage of cells with pSyn inclusions also positive for *Lamp5* in each cortical layer. Data in panels **e**, **f**, **i**, **j**, and m are presented as mean values +/- SEM with values for individual mice plotted (*n* = 12 mice). Source data are provided as a Source Data file.

We performed cell deconvolution on α-synuclein PFF-injected mice using the SpatialDecon^40^ algorithm to determine cell type proportion using the whole cortex and hippocampus 10X genomics single-cell dataset from Allen Brain Institute as the reference, which was subsampled specifically to each of our regions of interest^42^. Clear differences emerged between pSyn- and pSyn+ segments. In layer 5, the pSyn- segment showed enrichment of layer 5 PT, parvalbumin (Pvalb), and somatostatin (Sst) neurons, while the pSyn+ segment showed enrichment of L5 IT neurons (Fig. 6a, Supplementary Fig. 13). In layer 6, *Sncg*+ neuron types were less abundant in pSyn+ segments, while L6 IT neurons showed similar abundance in each segment, and L6b neurons were enriched in pSyn+ segments (Fig. 6b, Supplementary Fig. 13). Thus, we conclude that most inclusions in the cortex of the α-synuclein PFF model at this timepoint are formed in L5 IT, L6 IT, and L6b neurons, while PT and inhibitory neurons are less impacted.

To validate our deconvolution results, we performed cell typing with in situ hybridization-immunofluorescence spatially registered to the Allen Brain Atlas CCFv3. We first stained tissue with a broad excitatory neuron marker (*Slc17a7*), a broad inhibitory neuron marker (*Gad1*) and pSyn (Fig. 6c, Supplementary Fig. 14A). pSyn inclusions were almost exclusively located in *Slc17a7*+ neurons, while they were absent from *Gad1*+ neurons (Fig. 6d). To obtain more quantitative data on this localization, we performed cell detection and classification on all neurons in the cortex (Supplementary Fig. 15). In these sections, we confirmed that pSyn pathology was primarily in layer 5, with less pathology in layer 6, and minimal pathology in layers 2/3 (Fig. 6e). Across layers, almost all pSyn inclusions were in *Slc17a7*+ neurons, while no pSyn inclusions were identified in *Gad1*+ neurons (Fig. 6f).

We next sought to identify markers that could more selectively label IT and PT neurons. *Pcp4* is broadly expressed throughout the cortex (Supplementary Fig. 14b) with a preference for excitatory neurons, including both IT and PT neurons in layer 5 and several neuron types in layer 6^42^. *Rorb* is more selectively expressed, mostly in upper layer 5, bordering layers 2/3 (Supplementary Fig. 14b) and is expressed most highly in IT neurons, with less expression in PT neurons^42^. Most pSyn inclusions in layer 5 were in *Pcp4* + /*Rorb* + (IT) neurons, while neurons in layer 6 lacked *Rorb* staining (Fig. 6g, h). Quantitatively, layers 2/3 and 5 showed *Rorb* expression in the majority of neurons with pSyn inclusions (Fig. 6i). Broad *Pcp4* expression (excitatory) marked most neurons with pSyn inclusions across layers (Fig. 6j).

*Lamp5* is primarily expressed in layer 5 PT neurons and layers 2/3 IT neurons, with lower expression in layer 5 IT neurons (Supplementary Fig. 14c)^42^. Looking particularly in layer 5, pSyn inclusions do not appear in neurons expressing high *Lamp5* (PT, Fig. 6k), although a small amount of *Lamp5* can be observed in some inclusion bearing neurons (Fig. 6k, l). Though there are few pSyn inclusions in layers 2/3, some of those are in *Lamp5*+ neurons (L2/3 IT), while very few pSyn inclusions in layer 5 are positive for *Lamp5* (PT) (Fig. 6m), and these are in lower-expressing *Lamp5* neurons. It is possible that *Lamp5* low and high expressing neurons are distinct neuron populations, or that pSyn inclusion burden results in a phenotypic downregulation of *Lamp5* expression. To distinguish between these two possibilities, we quantified *Lamp5* low-expressing cells across hemispheres. The majority of Lamp5 low-expressing cells do not have pSyn inclusions, and there is no difference in the number of these cells across hemispheres (Supplementary Fig. 14d), suggesting that *Lamp5* low-expressing cells represent a cell population, distinct from L5 PT neurons. Together, these experiments support the idea that pSyn inclusions primarily impact layer 5 IT neurons in the α-synuclein PFF model.

### LBs are enriched in layer 5 intratelencephalic neurons in PD cortex

**Fig. 7:**
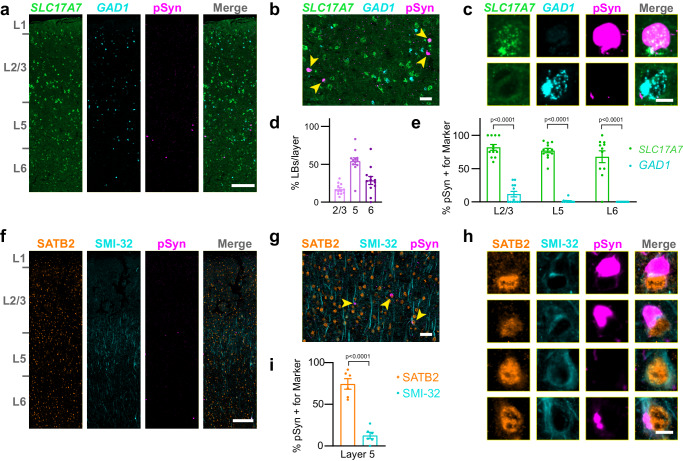
LBs are enriched in layer 5 intratelencephalic neurons in PD cortex. **a** A whole slice through human cingulate cortex stained for *SLC17A7*, *GAD1*, and pSyn. Scale bar = 250 µm. **b** A zoomed in view of layer 5 with yellow arrowheads denoting neurons with pSyn inclusions. Scale bar = 50 µm. (**c**) Examples of single cells positive for *SLC17A7*, *GAD1*, and/or pSyn. Scale bar = 10 µm. **d** Quantification of the percentage of LBs in each cortical layer as a percentage of the total inclusions shows the highest inclusion burden in L5, followed by L6 (*n* = 12 cases). **e** Quantification of the percentage of cells with LBs that were also positive for either *SLC17A7* or *GAD1* in each cortical layer. *p*-values are displayed from a mixed-effects analysis with Sidak’s multiple comparisons test to compare *SLC17A7* and *GAD1* positive neurons (*n* = 12 cases). **f** A whole slice through human cingulate cortex stained for SATB2, SMI-32, and pSyn. Scale bar = 250 µm. **g** A zoomed in view of layer 5 with yellow arrowheads denoting neurons with pSyn inclusions. Scale bar = 50 µm. **h** Examples of single cells positive for SATB2, SMI-32, and/or pSyn. Scale bar = 10 µm. **i** Quantification of the percentage of cells with LBs that were also positive for either SATB2 or SMI-32 in each cortical layer. *p*-value is displayed from a two-tailed *t*-test to compare SATB2 and SMI-32 positive neurons (n = 6 cases). Data in panels **d**, **e**, and **I** are presented as mean values +/- SEM with values for individual cases plotted. Source data are provided as a Source Data file.

To assess the distribution of LBs in PD, we carried out analogous staining and cell class quantification experiments (Supplementary Fig. 16). We first assessed broad markers of excitatory and inhibitory neuron types, *SLC17A7* and *GAD1*, by in situ hybridization-immunofluorescence (Fig. 7a–c). LBs in this tissue showed a similar distribution to that seen in α-synuclein PFF-injected mice, with inclusions primarily in layer 5, with lower burden in layers 6 and 2/3 (Fig. 7d). Inclusions were also almost exclusively in excitatory *SLC17A7*+ neurons across layers, with few inclusions found in inhibitory *GAD1*+ neurons (Fig. 7e).

To further assess the balance of LBs in IT versus PT neurons in PD, we stained tissues with antibodies recognizing SATB2, a nuclear protein expressed broadly in excitatory neurons, and neurofilament heavy chain (SMI-32), a protein enriched in L5 PT neurons (Fig. 7f). While SATB2 was distributed across cortical layers, SMI-32 showed an enrichment in layer 5. Inclusions were almost all in SATB2+ (excitatory) neurons, while SMI-32 positive neurons (PT) rarely had LBs (Fig. 7g, h). Cell classification identifying SATB2 + /SMI-32- compared to SATB2+/SMI-32+ neurons showed that around 75% of LBs were only positive for SATB2, while around 12% of neurons were also positive for SMI-32 (Fig. 7i), consistent with previous examination of SMI-32/LB co-localization in the superior temporal sulcus of DLB^19^. Together, these results support that in PD, layer 5 IT neurons are predominantly impacted by LBs.

### Single-cell spatial transcriptomics confirms the vulnerability of L5 IT and L6b neurons in mice

**Fig. 8:**
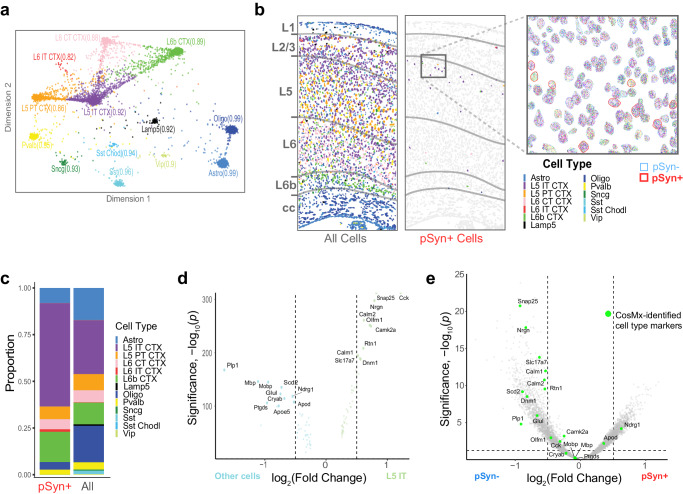
Single-cell spatial transcriptomics confirms the vulnerability of L5 IT and L6b neurons in mice. **a** Flightpath plot from InSituType-based cell type identification. Each cell is placed based on posterior probability of belonging to the assigned cell type (e.g., the distance from the centroid of the cell type cluster reflects confidence of cell type call). **b** Cell segmentation overlayed with cell type colors from representative fields-of-view from the cortex of one mouse brain. The first image shows all cells. The second image plots only pSyn+ cells, with other cells in gray and cortical layer outlines overlaid. The cutout displays all cells in the highlighted area with each colored dot representing a spatially resolved individual transcript within that cell (red outline indicates pSyn+ cells. **c** Barplot displaying the proportion each cell type from pSyn+ cells (pSyn) all detected cells (All). **d** Volcano plot of differential gene expression between cells identified as L5 IT compared to other cell types as determined by two tailed mixed effects linear regression with multiple test correction using FDR. **e** Volcano plot of genes differentially expressed between pSyn and NeuN segments in GeoMx experiments (as in Fig. 4b), except all genes are gray except those identified by CosMx as L5 IT cell type markers as determined by two tailed mixed effects linear regression with multiple test correction using FDR. Many L5 IT neuron markers are differentially expressed, but most of the DEGs are not L5 IT neurons markers identified in the current study.

Many genes are differentially expressed by cell type^41^, and it follows that cell types are best defined by a combination of many genes. To enable more comprehensive assessment of cell type, we next used single-cell spatial transcriptomics to clearly identify cell types with pSyn aggregates and delineate gene expression differences in those cells that were due to cell type in contrast to those that were due to cells developing inclusions. Mice injected with α-synuclein PFFs were aged 3 months post-injection (MPI) and brain tissue was mounted on slides for characterization using the 1000-plex Mouse Neuroscience Panel from nanoString on the CosMx instrument^43^. Data was collected from 6 fields of view spanning all 6 cortical layers of the MOs and corpus callosum in 3 mice (Supplementary Fig. 17). Three morphology markers were used to guide cell segmentation (Histone H3: nucleus, 18s rRNA: cytoplasm, GFAP: astrocytes). Following collection of mRNA transcript imaging data, tissue was stained for pSyn, and this stain was used to identify those cells with and without pSyn pathology. Cellpose neural network models^44^ were used for nuclear and cytoplasm cell segmentation. In total, 9941 individual cells were segmented, containing an average of 341 total transcripts per cell, with 171 unique genes per cell. Using the InSituType algorithm recently validated for CosMx data^45^, we were able to confidently identify 13 distinct cell types (Fig. 8a). The advantage of InSituType over other dimension reduction algorithms is that it provides a statistical estimate of the confidence of each cell-type cluster and each cell’s strength of relationship with each cell-type cluster visualized proportionally in a flightpath plot (Fig. 8A). Cells that were clustered based on transcriptional similarity were mapped back into anatomical space (Fig. 8b, Supplementary Fig. 17), demonstrating expected anatomical distributions. When these cells were subsampled for pSyn-positive cells (Fig. 8b), we confirmed that L5 IT and L6b neurons are enriched in aggregate bearing cells and L5 PT, Pvalb, and Lamp5 neurons are enriched in non-aggregate bearing cells (Fig. 8c).

In both human and mouse GeoMx data, we were able to identify known cell type genes that were differentially-expressed between pSyn and NeuN segments (Fig. 5c, d). We expect that we have differentially enriched for specific neuron populations with the pSyn- and pSyn+ segmentation, and we sought to further define the impact of cell type on the LAMDA signature identified. We first assessed DEGs that distinguished the L5 IT neuron population from other cells in the CosMx data (Fig. 8d). We then identified those likely cell type marker genes in our mouse GeoMx DEG plot. We found that many cell-type genes were enriched among DEGs but constituted a small fraction of the overall DEG pattern (Fig. 8e).

We next used a published list of genes enriched in specific cell types^41^ to examine cell-type selective DEGs between pSyn- and pSyn+ segments in different brain regions (Supplementary Fig. 18). Of the 8065 genes detected in our human dataset, 228 (2.6%) were identified as cell type selective. Of the 2042 DEGs in human cingulate cortex, 30 (1.5%) were cell-type selective genes (Supplementary Fig. 18a, b), suggesting that comparing neurons with and without Lewy bodies slightly depletes cell type marker genes. We found similar results from the mouse GeoMx experiment, where 228/9035 (2.5%) of detected genes were cell type selective, but only 49/4710 (1.0%) of DEGs were cell type markers (Supplementary Fig. 18c, d). Together, these results show that while cell type markers are present in the LAMDA signature, they represent a small portion of the overall genes. This is perhaps not surprising given that the conserved DEGs were compared across brain region and cortical layer, so remaining DEGs are more likely to be independent of specific cell types.

## Discussion

The goals of this study were to identify cortical neuron types vulnerable to developing α-synuclein inclusions, and to identify molecular signatures associated with the presence of those inclusions. Soon after the identification of α-synuclein as a main component of LBs^46^, staging studies showed that cortical areas are progressively impacted in PD^16^, and cortical LBs are associated with impaired cognitive performance^4–8^. In the SNc, the ventral tier is most vulnerable to degeneration^11,12^, especially *SOX6-AGTR1*+ neurons^13^. Yet, information about the molecular identity of vulnerable cortical neurons is lacking. Previous studies have reported that pathology develops first in deep layers of the cortex^16,17,47^ and disproportionately affected unmyelinated projection neurons, while myelinated projection neurons (presumably PT) and local circuit neurons, including inhibitory neurons, are protected^16,47^. These original observations are consistent with our data showing sparing of inhibitory neurons and PT neurons. Interestingly, IT neurons may be selectively vulnerable even in the absence of Lewy pathology. Characterization of the presupplementary motor area in early PD brains with minimal cortical pathology found that cortico-cortical projection neurons were selectively lost in PD, while large SMI-32+ corticospinal neurons were retained^48^.

Excitatory neurons have also been shown to be selectively vulnerable in the α-synuclein PFF model^49^. We expand this work to show that it is selectively L5 IT and L6b neurons that are vulnerable. The direct connection of L5 IT neurons to the striatum may be one reason for this vulnerability, but L5 PT neurons also send collateral axons to and through the striatum^50^, giving them the opportunity to be exposed to α-synuclein PFFs as well. Further, the conservation of cell-type vulnerability in the α-synuclein PFF model and PD suggest that both connectivity and cell intrinsic vulnerability characteristics may be conserved.

We also identified conserved molecular changes in aggregate-bearing neurons in both mice and PD that we have designated the LAMDA signature. This examination of changes within α-synuclein inclusion-bearing neurons demonstrates that these neurons are in a fundamentally altered state where synaptic connections and several cellular functions have been deprioritized and maintenance of DNA integrity or the initiation of apoptosis are prioritized. The LAMDA signature recapitulates the hallmarks of neurodegenerative disease^51^ (pathological protein aggregation, synaptic defects, aberrant proteostasis, cytoskeletal abnormalities, altered energy homeostasis, DNA and RNA defects, neuronal cell death, inflammation), suggesting that even within brains experiencing neurodegeneration, cells bearing aggregates are impacted in a cell-autonomous manner.

Synapses are critical for functional connectivity of neurons, and synapses are impaired early in PD. Dopaminergic terminal loss precedes neuron loss in PD^52,53^, and this loss of terminals can be used clinically to diagnose PD and monitor progression^54^. It has been proposed that this synaptic vulnerability is related to the burden of maintaining distant, metabolically demanding presynaptic terminals^55^. Synaptic vulnerability is also seen in glutamatergic neurons. In primary neuron culture, α-synuclein PFF-induced pathology leads to a broad downregulation of synaptic genes and proteins, and a corresponding loss in synapses^32,34^. In the LAMDA signature, there is broad downregulation of presynaptic genes, especially those involved in the synaptic vesicle cycle (*SNAP25, STXBP1, SYT1, VAMP2, CPLX2, NSF, DNM1*). There is also downregulation of postsynaptic genes including NMDA (*GRIN1*) and AMPA (*GRIA1*) receptors, with the notable exception of some metabotropic glutamate receptors (*GRM7* is elevated in neurons with LBs).

Together with the loss of synapses in PD comes a disruption to axonal transport and the associated cytoskeleton. Previous research has shown that Lewy neurites in the axon may impair axonal cytoskeleton integrity and limit the axonal transport^56^ essential for trophic support and endolysosomal maturation^57^. There is a reduction in cytoskeleton transport proteins early in PD^53^, preceding the loss of TH staining, and α-synuclein pathology has previously been linked to modulation of the actin cytoskeleton^58^. LAMDA includes downregulation of several cytoskeletal genes (*TUBB4B, NEFL, SEPTIN7, CFL1*) and cytoskeleton transport genes (*KIF5A*), consistent with a reduction of synapses and axons in PD.

Mitochondria have also been heavily implicated in PD pathogenesis. α-synuclein fibrils can directly interact with mitochondria, and dysmorphic mitochondria are found within α-synuclein inclusions^34,59,60^. This sequestration of damaged mitochondria could be the cause of decreased mitochondrial respiration in primary neurons treated with α-synuclein PFFs^34^. Loss-of-function mutations in PINK1 or Parkin, which control mitophagy, can lead to familial PD^61,62^ in the absence of Lewy pathology^63^. Interestingly, *PINK1* expression is decreased in LAMDA, which could be linked to the general downregulation of mitochondrial genes, or it could be a downstream result of LB toxicity. Neurotoxins targeting complex I of the mitochondria, such as MPP+ (1-methyl-4-phenylpyridinium), can induce Parkinsonism by killing dopaminergic neurons^64^ and are therefore used to model PD in rodents^65^. Loss of mitochondrial complex I activity is also sufficient to drive progressive dopaminergic degeneration^66^. Interestingly, the gene deleted in these mice, *Ndufs2*, is downregulated in pSyn neurons in mice, while a complex I gene whose knockout did not induce degeneration, *Ndufs4*, was not. It is unclear why oxidative phosphorylation pathways are downregulated in LAMDA. It is possible that metabolic demand on neurons with reduced axons and synapses is reduced, or disruption of mitochondria may precede neuron death, since the mitochondria is intimately linked to the apoptotic process^67^.

Ubiquitination has many functions, but in neurodegeneration it is typically associated with degradation, either via the proteasome, autophagy, or related mitophagy. Protein aggregates, including LBs themselves are highly ubiquitinated^68^. This could be a failed attempt to degrade the misfolded proteins, or a mechanism for clustering them together for further processing^69^. Aggregates are not the only proteins ubiquitinated in neurodegeneration. In particular, ubiquitination of mitochondria is a critical step in mitophagy. Parkin, an E3 ligase, mediates this process after phosphorylation by PINK1. This process is highly controlled by deubiqutinases, including USP30^70^. For that reason, upregulation of PINK1-Parkin or inhibition of USP30 have been pursued as a potential therapeutics for PD^71^. Interestingly, we find downregulation of *PINK1* and increase in *USP30* (in human, not detected in mouse) and other deubiqutinases (*USP38, USP42*) in LAMDA, suggesting the validity of this therapeutic approach for idiopathic PD. Consistent with the reduced machinery for mitophagy, lysosomal proteases, including *CTSA, CTSB, CTSD* are downregulated. However, it is important to note that the changes seen in LAMDA may be protective for PD as well. An efficient method to minimize circuit disruption and reduce cell-to-cell spread of pathology may be to pull back from the network by reducing synaptic connections, and if necessary, kill the cell to save the circuit.

Along these same lines, an important question for the field is whether anti-apoptotic treatments would be effective in neurodegenerative diseases or simply modify the inevitable dysfunctional molecular changes that culminate in cell death^72^. We see activation of pathways in LAMDA that have been previously implicated in apoptosis in neurodegenerative disorders^72^. We see an increase in pro-apoptotic factors *BAX* and *BAK1* in human (involved mitochondrial outer membrane permeabilization^67^). Due to the role of mitochondria in cell death^67^, we considered a known mitochondria-apoptotic pathway and examine gene expression of that pathway in LAMDA. Mitochondrial outer membrane permeabilization leads to mitochondrial DNA release, which interacts with cGAS (*Cgas*, up in mouse pSyn) which signals through STING to IRF3 and NF-κB (*Nfkb1*, up in mouse pSyn) and leads to interferon expression (*Ifnar1* up in mice, *IFNAR2* up in human). We also see induction of complement and cytokine pathways in LAMDA, which is consistent with previous research which has found complement proteins in SNc neurons in PD^73–75^. Thus, gene expression profiles in LAMDA support a potential mitochondria-mediated apoptosis in these neurons. However, there is also an increase in the anti-apoptotic gene *Bcl2* in mice, suggesting that there may be a mixture in inclusion-bearing neurons of staving off apoptosis and promoting it.

The induction of these pathways is consistent with the progressive death of neurons bearing inclusions. While it is difficult to track the trajectory of these neurons in humans, live imaging of transgenic mice injected with α-synuclein PFFs has shown the progressive death of cortical neurons bearing inclusions^33,76^. Apoptotic pathways can be induced by DNA damage, ER stress, oxidative stress, and cytoskeleton alterations. α-Synuclein inclusions are related to the presence of double-strand DNA breaks and the molecular machinery associated with repair of those breaks (γH2AX) both in mouse and human^77^. This genome instability^78^ may be due to oxidative stress in PD neurons^79^. Several components of the DNA repair pathway are upregulated in LAMDA, including *H2AX*, and genes involved in nucleotide excision repair *GTF2H1, GTF2H3, RPA2*. Manipulation of this pathway has also been found to be protective in *C. elegans* with α-synuclein overexpression^80^.

While no other study has profiled the inclusion status of neurons, recent publications have used RNAseq to profile PD neurons, primarily in the SNc. While some of these studies primarily focused on lost neuron types^13,14^, more recent studies have also examined gene expression changes in PD neurons^15^. Interestingly, there are some conserved gene signatures between PD SNc neurons, and the cortical LAMDA signature. Synaptic genes (*SNAP25, SYT1, NSF, CACNA1A, GRIK2, GRIA4*), cytoskeletal genes (*NEFL, TUBA1B*), and monogenic PD genes (*SYNJ1, UCHL1*) are downregulated in both datasets. However, not all the SN neurons would be expected to have LBs, and we see interesting differences in these datasets as well. While HSF1 activation is present in the SNc, we see the opposite in LB-bearing neurons (downregulation of *HSPA8, HSPA4L, HSP90AA1, HSPH1*). We also see some conserved increase in metallothioneins (*MT1E, MT1M*), but a contrasting downregulation in others (*MT3*).

There are fewer studies of RNAseq in PD cortex. One study, which profiled excitatory neurons in L5 and L6 of posterior cingulate^20^ identified some changes that were conserved in our dataset, including decreased *OXR1*, which responds to oxidative stress, a decrease in cytoskeletal transport gene *KIF21A*, a decrease in synaptic transmission genes, and decreased proteasomal subunits. However, some gene changes were not replicated, which may reflect changes in neurons without LBs (decrease in *FGF9*). An additional study, which performed bulk and scRNAseq from control and PD cingulate cortex^21^ did not describe specific gene expression changes deeply, but found that the number of DEGs was highest in excitatory neurons, and *PINK1* was downregulated in these neurons, consistent with excitatory neurons bearing LAMDA-related changes.

There is only one other study, to our knowledge, that has examined the transcriptional state of neurons bearing aggregates^81^. This study purified neurons with and without tau neurofibrillary tangles (NFTs) from the prefrontal cortex of Alzheimer’s disease (AD). There appears to be some conserved cellular vulnerability between LBs and NFTs, with L5 IT and L6b neurons identified in both studies. However, additional neurons, including a subset of inhibitory neurons, developed NFTs, while LBs did not appear in those neurons. The DEG profile was completely different in NFT neurons than in LAMDA. Synaptic genes were up in NFT neurons, down in LAMDA. Oxidative phosphorylation and mitochondrial dysfunction were not conserved across NFT neurons, and apoptosis genes were only modestly enriched. Specific genes highlighted as increased in NFT neurons (*HSP90AA1, APP, PRNP*) were all decreased in LAMDA. Additional results from snRNAseq experiments in AD are consistent with the contrasting signatures in AD and PD^82^. Several genes that are increased in AD are decreased in LAMDA (*ST6GALNAC6, BIN1, RAB3A*), although downregulation of metabolic genes is more consistent (*CYCS, NDUFA8* down in AD and LAMDA). These results suggest that while there may be some conservation in factors mediating vulnerability to NFTs and LBs, the cellular response to these two cytoplasmic aggregates is distinct. This distinct cellular response may give insight into why the genetic risk factors for AD and PD are different and also indicate that it may be difficult to find therapies that would be effective in both AD and PD.

There are several limitations to the current study. A primary limitation is the lack of single-cell resolution in the GeoMx data. We suspect that the heterogeneity in gene signatures, especially among neurons bearing inclusions, is due to a variety of cell states that is lost with the admixture of cells required to obtain sufficient sequence saturation in this study. We complement the GeoMx data with CosMx single cell spatial molecular imaging, which confirmed impacted cell types, but the genome coverage from this technique is limited. Future studies may identify a series of cell states in neurons bearing inclusions that can be aligned by pseudotime to reconstruct the trajectory neurons take to dealing with LB inclusions. A second limitation is that we focused on a handful of cortical regions. We suspect that the LAMDA signature may be conserved across cell types in different regions based on similar signatures being identified in SNc neurons in PD^15^ and a mouse model of PD^83^, however future studies will be necessary to determine conserved and disparate gene expression changes by cell type.

Leaps have been made recently understanding molecular profiles of cells in neurodegenerative disease. Our study expands on those findings, showing molecular changes in neurons with LBs in PD. We further show a conservation of gene expression profile in a mouse model of α-synucleinopathy and PD and have designated this conserved signature LAMDA. We expect LAMDA will provide researchers, including ourselves, the ability to probe our pathways of interest and understand how they are impacted in aggregate-bearing neurons. We also hope it will provide insight about which pathways are most amenable to therapeutic intervention, especially interventions targeting the onset of dementia in PD patients. Finally, we believe that this initial study will be a starting point for future studies of neuronal vulnerability across the brain and through disease progression.

## Methods

### Animals

All housing, breeding, and procedures/experiments were performed according to the NIH Guide for the Care and Use of Experimental Animals and approved by the Van Andel Institute Institutional Animal Care and Use Committee (IACUC). *Mus musculus* C57BL/6J mice were purchased from the Jackson Laboratory (000664; RRID:IMSR_JAX:000664) and housed at 22 °C and 50% humidity with 12-hour light cycles (lights on at 7 am, lights off at 7 pm). Both male and female mice were used and were 3-4 months old at the time of injection. Animals were aged 6–7 months old prior to sample collection. 5 male and 6 female mice were utilized in the GeoMx experiments and 1 female and 2 male mice were used for CosMx.

### Recombinant α-synuclein preparation

Purification of recombinant mouse α-synuclein and generation of α-synuclein PFFs was conducted as described elsewhere^84,85^. The pRK172 plasmid containing the mouse *Snca* sequence was transformed into BL21 (DE3) RIL-competent E. coli (Agilent Technologies Cat#230245). A single colony from this transformation was expanded in Terrific Broth (12 g/L of Bacto-tryptone, 24 g/L of yeast extract 4% (vol/vol) glycerol, 17 mM KH_2_PO_4_ and 72 mM K_2_HPO_4_) with ampicillin. Bacterial pellets from the growth were sonicated and the sample was boiled to precipitate undesired proteins. The supernatant was dialyzed with 10 mM Tris, pH 7.6, 50 mM NaCl, 1 mM EDTA overnight. Protein was filtered with a 0.22 µm filter and concentrated using Amicon Ultra-15 centrifugal filter units (Millipore Sigma Cat#UFC901008). Protein was then loaded onto a Cytiva HiLoad 26/600 Superdex 200 pg (Cytiva Cat#28989336) column and 5 mL fractions were collected. Fractions were run on SDS-PAGE and stained with InstaBlue (ApexBio B8226) to select fractions that were highly enriched in α-synuclein. These fractions were combined and dialyzed in 10 mM Tris, pH 7.6, 50 mM NaCl, 1 mM EDTA overnight. Dialyzed fractions were applied to the MonoQ column (GE Health, HiTrap Q HP 645932) and run using a linear gradient from 25 mM NaCl to 1 M NaCl. Collected fractions were run on SDS-PAGE and stained with Coomassie blue. Fractions that were highly enriched in α-synuclein were collected and dialyzed into DPBS. Protein was filtered through a 0.22 µm filter and concentrated to 5 mg/mL (α-synuclein) with Amicon Ultra Centrifugal Filters. Monomer was aliquoted and frozen at −80 °C. For preparation of α-synuclein PFFs, α-synuclein monomer was shaken at 1000 rpm and 37 °C for 7 days. Conversion to PFFs was validated by sedimentation at 100,000 x *g* for 60 minutes and by thioflavin T assay.

### Stereotaxic injections

All surgery experiments were performed in accordance with protocols approved by the IACUC of Van Andel Institute. α-Synuclein PFFs were vortexed and diluted with DPBS to 2 mg/mL and sonicated in a cooled bath sonicator at 9 °C (Diagenode Bioruptor® Pico; 10 cycles; setting medium; 30 seconds on, 30 seconds off). Mice were injected when 3-4 months old. Mice were deeply anesthetized with isoflurane and injected unilaterally into the right forebrain targeting the dorsal striatum (coordinates: +0.2 mm relative to Bregma, +2.0 mm from midline, −2.5 mm beneath the dura) Injections were performed using a 10 µL syringe (Hamilton 7635-01, NV) with a 34-gauge needle (Hamilton 207434, NV) injecting 5 µg α-synuclein PFFs (2.5 µL) at a rate of 0.4 µL/minute. After 3 months, mice were perfused transcardially with 0.9% saline and 4% paraformaldehyde (PFA), brains were removed and underwent overnight fixation in 4% PFA. After perfusion and fixation, tissues were processed into paraffin via sequential dehydration and perfusion with paraffin under vacuum (70% ethanol for 1 hour, 80% ethanol for 1 hour, 2 times 95% ethanol for 1 hour, 3 times 100% ethanol for 1 hour, 2 times xylene for 30 minutes, paraffin for 30 minutes at 60 °C, paraffin for 45 minutes at 60 °C). Brains were then embedded in paraffin blocks, cut into 6 µm sections and mounted on glass slides.

### Human brain tissue

All procedures were done in accordance with local institutional review board guidelines of the Banner Sun Health Research Institute. Written informed consent for autopsy and analysis of tissue sample data was obtained either from patients themselves or their next of kin. Tissues were selected based upon a high burden of Lewy pathology by immunohistochemical staining. Non-pathological cases were balanced by age, sex, and PMI. Human brain tissue was briefly thawed from −80 °C on ice before being transferred to ice-cold 10% neutral buffered formalin. Tissue was fixed for 24 hours before being processed into paraffin as noted for mouse tissues. Tissue was cut into 6 µm sections and mounted on glass slides.

### GeoMx spatial transcriptomics

Spatial transcriptomics was performed using the nanoString GeoMx® Digital Spatial Profiler. Sections were cut at 6 μm thickness and mounted on plus-charged slides (Epredia Colormark Plus CM-4951WPLUS-001). Slides were baked at 60 °C for 1 hour and stored at 4 °C in a vacuum sealed container containing desiccant for up to two weeks. All subsequent steps were performed using RNase-free conditions and DEPC treated water. Slides were de-paraffinized with 3 sequential 5-minute washes in xylenes, followed by 2 washes in 100% ethanol for 5 minutes, 1 wash in 95% ethanol, and 1 wash in 1x PBS. Target retrieval was performed in target retrieval reagent (10x Invitrogen 00-4956-58 EDTA pH 9.0) diluted to 1x in the BioGenex EZ-Retriever System for 10 minutes at 95 °C. Slides were then washed with 1x PBS for 5 minutes. Slides were then incubated in 0.1 μg/mL proteinase K (Invitrogen 25530-049) for 10 minutes at 37 °C and washed in 1x PBS for 5 minutes at room temperature. Slides were post fixed for 5 minutes in 10% neutral buffered formalin followed by two washes in NBF stop buffer (24.5 g Tris base and 15 g Glycine in 2 L DEPC water) for 5 minutes each and one wash in 1x PBS for 5 minutes. Slides were then incubated with hybridization probes (nanoString Cat# 121401103) diluted in Buffer R (provided in the GeoMx RNA Slide Prep FFPE- PCLN kit, catalog # 121300313) in a hybridization oven at 37 °C for 16-20 hours.

Following probe incubation, slides were washed with stringent washes (equal parts formamide and 4x SSC buffer) at 37 °C twice for 25 minutes each. Then slides were washed twice in 2x SSC buffer. Slides were incubated in 200 μL buffer W (provided in the GeoMx RNA Slide Prep FFPE- PCLN kit, catalog # 121300313) for 30 minutes and incubated in morphology markers (GFAP-488, Thermo 53-9892-82, RRID:AB_10598515, 1:400; pS129 α-synuclein (81A), BioLegend 825701, RRID:AB_256489, 1:1000; NeuN, Millipore ABN78, RRID:AB_10807945, 1:1000) at 4 °C overnight. Slides were washed 4 times in 2x SSC buffer for 3 minutes each wash. Slides were then incubated with secondary antibodies (GαRb 647, Thermo Scientific A21244, RRID:AB_2535812, 1:1000; GαIgG2a 594, Thermo Scientific A21135, RRID:AB_2535774, 1:1000) and nuclei marker Syto83 (Thermo Scientific S11364, 1:1000) in Buffer W for 1 hour at room temperature in a humidified chamber. Slides were washed 4 times in 2x SSC buffer for 3 minutes each and placed in the nanoString GeoMx® DSP instrument.

Syto83 immunofluorescence was utilized for autofocus of GeoMx imaging. Immunofluorescence for GFAP was used in identification of morphological markers to aid in fitting to the Allen Brain Institute’s mouse brain atlas. NeuN and pSyn were used for segmentation. Prior to region-of-interest (ROI) generation, the slide image was exported, and individual brains were registered to the Allen Brain Atlas CCFv3 (10.1016/j.cell.2020.04.007) using the mouse brain registration protocol. Images of registered brains were imported onto the DSP instrument and fit exactly to the slide image to enable accurate anatomical selection of ROIs. ROIs were generated using the polygon tool which aligned with cortical layer 5 or layer 6 of the anterior cingulate area (ACAd or ACAv), primary motor cortex (MOp), and secondary motor cortex (MOs). Each ROI was segmented into two areas-of-illumination (AOI): “pSyn + ” neurons positive for pS129 α-synuclein and NeuN or “pSyn-” neurons positive for NeuN and negative for pS129 α-synuclein. Probe identities in each segment were captured via UV illumination and movement to a 96-well plate.

### NGS library prep and sequencing

Illumina Novaseq 6000 was used for sequencing with a read length of 27 for both reads with reverse sequence orientation in the readout group plate information. Plates were dried down and rehydrated in 10 µL nuclease-free water, mixed and incubated at room temperature for 10 minutes. PCR was performed on samples as described in the nanoString GeoMx® DSP Readout User Manual using 2 µL PCR master mix, 4 µL primer from the correct wells, and 4 µL resuspended DSP aspirate. KAPA beads (KAPA Pure beads, Roche Cat# 07983298001) were warmed to room temperature for 30 minutes. Libraries were pooled and KAPA beads were added to each pool at a 1.2X ratio to the final pool volume. Two KAPA bead clean ups were performed and pooled libraries were eluted in 24 µL elution buffer. Negative and positive control pools were eluted into 10 µL elution buffer. Quantity of the pools were assessed using the QuantiFluor® dsDNA System (Promega Corp.). Pools were diluted to 5 ng/µL and quality and size are assessed using Agilent DNA High Sensitivity chip (Agilent Technologies, Inc.) on the Bioanalyzer. Sequencing was performed at 100 reads/µm^2^. Paired end 50 base paired sequencing. Base calling was done by Illumina RTA3 and output was demultiplexed and converted to fastq format with bcl2fastq v1.9.0. Fastq files were then converted to Digital count conversion files (DCC) using GeoMx NGS Pipeline v2.3.3.10. Sequencing reads were trimmed to 27 bp to reflect GeoMx probe length.

### CosMx spatial molecular imaging

The sample processing, staining, imaging, and cell segmentation for CosMx approach were performed as previously described^43^. Publicly available protocols were followed for tissue staining and probe acquisition (https://university.nanostring.com/page/document-library). Briefly, tissue sections were placed to VWR Superfrost Plus Micro Slide (Cat# 48311-703) for optimal adherence. Slides were then dried at 37 °C overnight, followed by deparaffinization, antigen retrieval and proteinase mediated permeabilization. 1 nM RNA-ISH probes were applied for hybridization at 37 °C overnight. Markers for morphology and cell segmentation were added: Histone H3: nucleus, 18 s rRNA: cytoplasm, GFAP: astrocytes. After stringent washing, a flow cell was assembled on top of the slide and cyclic RNA readout on CosMx was performed using a 16-digit encoding strategy. Following cycles, pSer129 α-synuclein was stained to mark for Lewy-like pathology (81 A, BioLegend 825701, RRID:AB_256489, 1:400) with fluorescent secondary antibody (GαM IgG2a, Thermo Scientific A21241, RRID:AB_2535810, 1:400). Two 0.7 mm × 0.7 mm fields of view (FOVs) were selected for data collection in each of the three mouse brains. FOVs were centered on secondary motor cortex. The CosMx optical system has an epifluorescent configuration based on a customized water objective (13×, NA 0.82), and uses widefield illumination, with a mix of lasers and light-emitting diodes (385 nm, 488 nm, 530 nm, 590 nm, 647 nm) that allow imaging of DAPI, Alexa Fluor-488, Atto-532, Dyomics Dy-605 and Alexa Fluor-647, as well as removal of photocleavable dye components. The camera was a FLIR BFS-U3_200S6M-C based on the IMX183 Sony industrial CMOS sensor (pixel size 180 nm). A 3D multichannel image stack (9 frames) was obtained at each FOV location, with the step size of 0.8 µm. Registration, feature extraction, localization, decoding of the presence individual transcripts, and machine-learning based cell segmentation (developed upon Cellpose^44^ were performed as previously described^43^. The final segmentation mapped each transcript in the registered images to the corresponding cell, as well as to subcellular compartments (nuclei, cytoplasm, membrane), where the transcript is located.

### In situ hybridization-immunofluorescence

In situ hybridization was performed with RNAscope® Multiplex Fluorescent Reagent Kit v2 (ACD, Cat #323270) using recommended conditions and supplied reagents. Paraffin-embedded tissue was freshly sectioned and dried. When not used immediately, slides were vacuum-sealed and stored at 4 °C. Slides were baked in a dry oven for 1 hour at 60 °C and used within one week. Slides were de-paraffinized with 2 sequential 5-minute washes in xylenes, followed by 2 washes in 100% ethanol for 2 minutes. Slides were then dried for 5 minutes at 60 °C. For autofluorescence quenching in human brain tissue, slides were incubated in 0.1 M Tris in a humidified tray for 3-5 days under a broad-spectrum LED lamp (Higrow LED, BL-E150A and King LED 3000 watt) until autofluorescence was diminished. Slides were treated with hydrogen peroxide for 10 minutes at room temperature and washed two times with distilled water. Target retrieval was performed in target retrieval reagents in the BioGenex EZ-Retriever System for 15 minutes at 99 °C. Slides were then washed with distilled water for 15 seconds and transferred to 100% ethanol for 3 minutes before being dried for 5 minutes at 60 °C.

Slides were incubated in protease plus in a humidified tray in a hybridization oven (Boekel Scientific 240200) oven for 30 minutes at 40 °C. Slides were washed 2 times with distilled water. RNAscope® probes were added to slides and incubated for 2 hours at 40 °C. The following probes were used: *Slc17A7* (Ms:416631, Hu:415611)*, Gad1* (Ms: 400951, Hu:404031)*, Pcp4* (Ms:402311), *Rorb* (Ms:444271), and *Lamp 5*(Ms:451071). Slides were washed twice for 2 minutes with wash buffer and incubated in Amp 1 for 30 minutes at 40 °C. The wash and Amp incubation was repeated for Amp 2 and Amp 3, except Amp 3 was only incubated for 15 minutes. Slides were washed twice for 2 minutes with wash buffer and incubated in HRP-C1 for 15 minutes at 40 °C. Slides were washed twice for 2 minutes with wash buffer and incubated in Opal 520 (Perkin Elmer FP1487A) or vivid 520 (ACDbio 323271) for 30 minutes at 40 °C, washed twice for 2 minutes with wash buffer, incubated in HRP blocker for 15 minutes at 40 °C, and washed twice for 2 minutes with wash buffer. The HRP, Opal dye, and HRP blocker steps were repeated for HRP-C2 using Opal 570 (Akoya Biosciences, OP001003) or vivid 570 (ACDbio, 323272). Following the final wash, the slides were further processed for immunofluorescence using the protocol described below but picking up starting after microwave antigen retrieval.

### Immunohistochemistry/Immunofluorescence

Slides were de-paraffinized with 2 sequential 5-minute washes in xylenes, followed by 1-minute washes in a descending series of ethanols: 100%, 100%, 95%, 80%, 70%. Slides were then incubated in deionized water for one minute prior and transferred to the BioGenex EZ-Retriever System where they were incubated in antigen unmasking solution (Vector Laboratories; Cat# H-3300) and microwaved for 15 minutes at 95 °C. Slides were allowed to cool for 20 minutes at room temperature and washed in running tap water for 10 minutes. Slides were incubated in 7.5% hydrogen peroxide in water to quench endogenous peroxidase activity (this step was skipped for immunofluorescence). Slides were washed for 10 minutes in running tap water, 5 minutes in 0.1 M Tris (diluted from 0.5 M Tris made from Tris base and concentrated hydrochloric acid to pH 7.6), then blocked in 0.1 M Tris/2% fetal bovine serum (FBS) for 15 minutes or more. Slides were incubated in primary antibody in 0.1 M Tris/2% FBS in a humidified chamber overnight at 4 °C (SMI-32, BioLegend 801702, RRID:AB_2715852, 1:250; SATB2 (Abcam ab92446, RRID:AB_10563678, 1:250); pS129 α-synuclein (81A, BioLegend 825701, RRID:AB_256489, 1:1000).

For immunohistochemistry, primary antibody was rinsed off with 0.1 M tris for 5 minutes, then incubated with goat anti-rabbit (Vector BA1000, RRID: AB_2313606) or horse anti-mouse (Vector BA2000, RRID: AB_2313581) biotinylated IgG in 0.1 M tris/2% FBS 1:1000 for 1 hour for immunohistochemistry. Biotinylated antibody was rinsed off with 0.1 M tris for 5 minutes, then incubated with avidin-biotin solution (Vector PK-6100, RRID: AB_2336819) for 1 hour. Slides were then rinsed for 5 minutes with 0.1 M tris, then developed with ImmPACT DAB peroxidase substrate (Vector SK-4105, RRID: AB_2336520) and counterstained briefly with Harris Hematoxylin (Fisher 67-650-01). Slides were washed in running tap water for 5 minutes, dehydrated in ascending ethanol for 1 minute each (70%, 80%, 95%, 100%, 100%), then washed twice in xylenes for 5 minutes and coverslipped in Cytoseal Mounting Media (Fisher, Cat# 23-244-256). Slides were scanned into digital format on an Aperio AT2 microscope using a 20x objective (0.75 NA) into ScanScope virtual slide (.svs) files.

For immunofluorescence, secondary antibodies were incubated on slides in a the dark for 3 hours at room temperature or overnight at 4 °C: GαM IgG1 488 (Invitrogen A21121, RRID:AB_2535764, 1:1000), GαRb 546 (Invitrogen A11010, RRID:AB_2534077, 1:1000), GαIgG2a 647 (Invitrogen A21241, RRID:AB_2535810, 1:1000). Slides were rinsed twice for 5 minutes in 0.1 M Tris in the dark. Human brain tissue was incubated in Sudan Black (0.3% Sudan Black B (Sigma, Cat# 199664) in 70% ethanol) for 10–120 seconds until autofluorescence was quenched. This step was not performed on RNAscope® slides due to pre-stain autofluorescence quenching. Slides were washed for 5 minutes in 0.1 M Tris and mounted with coverglass in ProLong gold with DAPI (Invitrogen, Cat#P36931). Fluorescent slides were imaged at 20x magnification on a Zeiss AxioScan 7 microscope.

### Cell detection

Stained slides were scanned on a Zeiss AxioScan 7 at 20x magnification and imported into QuPath v0.2.3 (RRID: SCR_018257) or newer for analysis. Individual cells were identified using the Cell Detection feature that allows detection based on DAPI stain intensity. Cell detection parameters such as background radius, sigma, and threshold were adjusted to optimize cell detection across all brain sections. For mouse tissue, brains were registered to the Allen Brain Atlas CCFv3, and all cells were quantified in the ACAv, ACAd, MOs, and MOp in each cortical layer. One tissue section from each mouse was used for quantification and data points are representative of one mouse. For human tissue, cortical layers were manually annotated and all cells within those cortical layers were classified and quantified as noted below.

### Cell classification

After cell detection, cell classification was completed in QuPath using the object classification feature. QuPath was trained on a subset of annotations to distinguish between different cell types based on signal intensity. To train the classifier, cells were marked as positive or negative for each marker. The training process was repeated for each marker of interest and a composite classifier was created to identify cells positive for multiple markers. Once the classifier was sufficiently accurate, the composite was loaded onto the entire brain image to classify all detected cells. Each cell type was given a unique 6-digit HTML color code. The investigators were blinded to expected outcomes.

### Mouse brain registration

Images were registered to the Allen Brain Atlas CCFv3 using a modified version of the QUINT workflow^36^. An RGB image of each section was exported from QuPath as a PNG, downsampled by a factor of 12, to use for spatial registration in QuickNII^37^ (RRID:SCR_016854). A segmentation image was created by exporting a color-coded image of classified cells by category on a white background to use as the segmentation input in Nutil (RRID:SCR_017183). Brain images were uploaded in Filebuilder and saved as an XML file to be compatible with QuickNII. Following the spatial registration of the mouse brain sections to the Allen Mouse Brain Atlas CCFv3 in QuickNII, a JSON file was saved for use in VisuAlign (RRID:SCR_017978). Brain sections were imported into VisuAlign to fine tune the registrations to match regions of interest. Anchor points were generated in the atlas overlay and moved to the corresponding location on the brain section via non-linear transformations. Markers were placed around the contour of the brain section first with markers refining the inner structures applied second. Final alignments were exported as FLAT and PNG files for use in Nutil^86^.

Nutil was used for the quantification and spatial analysis of the identified cell types in specific regions of the mouse brain. Individual classes were identified for quantification via their HTML color code assigned in QuPath. Nutil generated object counts from each individual classification within each region of the Allen Mouse Brain Atlas using the registration from QuickNII and VisuAlign. Regions of interest, Layers 1, 2/3, 5, 6a, and 6b of the dorsal anterior cingulate area (ACAd), ventral anterior cingulate area (ACAv), primary motor area (MOp), and the secondary motor area (MOs), were extracted from the Nutil output using a custom R code (RRID: SCR_001905, 10.5281/zenodo.10650925). Furthermore, the dorsal anterior cingulate area (ACAd) and the ventral anterior cingulate area (ACAv) data points were combined (ACA), as well as Layer 6a and Layer 6b for the MOp, MOs, and ACA.

### Statistics & reproducibility

#### Quality control

In the mouse GeoMx experiment, 124 segments were collected in total from 11 mice, and 21 of these segments failed Quality Control (QC) analysis and were removed from the dataset. One segment was removed for sequence stitching <80% and aligned sequence reads <75%. Five additional segments were removed for aligned sequence reads <75% alone, and four segments were removed for sequence saturation <50%. An additional 11 segments were removed for <3% of genes being detected above the Limit of Quantification (LOQ). Of the 20175 gene targets contained in the GeoMx Mouse Whole Transcriptome Atlas, 9035 were included in the downstream analyses following QC analysis. 1 gene target was removed as a global outlier (Grubbs test, P < 0.01), and 7 were removed as local outliers (Grubbs test, *P* < 0.01). 211 genes were detected below the limit of quantification and were removed. Of the remaining gene targets, 9,031 gene targets were detected above the LOQ in at least 10% of segments, and therefore were selected for further analysis. An additional 4 gene targets were identified as interesting a priori and were retained in the study for additional analysis. Detailed QC parameters are described in our mouse analysis https://github.com/Goralsth/Spatial-transcriptomics-reveals-molecular-dysfunction-associated-with-cortical-Lewy-pathology (10.5281/zenodo.10732492) Sample size was determined based on pilot data. No statistical method was used to determine sample size. Experiments did not involved multiple experimental conditions.

In the human GeoMx experiment 238 segments were collected in total from 8 human PD/PDD/DLB cingulate cortex samples (*n* = 6 male, *n* = 2 female). The Human GeoMx data was assessed on the same parameters as the mouse tissue for Quality Control (QC). In total 63 segments failed QC and were removed from the dataset. 33 samples were removed for low sequence saturation, and 6 segments were removed for low area. An additional 24 segments were removed for <5% of genes being detected above the Limit of Quantification (LOQ). Of the 18,815 gene targets present in the GeoMx Human Whole Transcriptome Atlas, 8,601 gene targets were included in the downstream analyses following QC. 9 gene targets were removed as local outliers (Grubbs test, *P* < 0.01). 138 genes were detected below the limit of detection and were removed. Of the remaining gene targets, 8,595 gene targets were detected in at least 10% of segments. 6 gene targets were identified as interesting a priori and were retained in the study for additional analysis. Detailed QC parameters are described in our human analysis pipeline https://github.com/Goralsth/Spatial-transcriptomics-reveals-molecular-dysfunction-associated-with-cortical-Lewy-pathology (10.5281/zenodo.10732492). Sample size was determined based on pilot data. No statistical method was used to determine sample size. Experiments did not involved multiple experimental conditions.

#### Normalization

To account for systematic variation between AOI’s, we normalized each count by the 75th percentile (Q3) of expression for each AOI. To ensure normalization and Quality Control (QC) procedures were accurate, we compared each AOI’s Q3 value to its limit of quantification. Normalization and QC procedures were determined to be adequate for robust downstream analysis.

#### Differential expression

Differential expression of gene targets in the GeoMx experiments was determined via a mixed effects linear regression model. The regression model implemented was as follows:

1 $${{{{{\rm{y}}}}}}={{{{{\rm{gene}}}}}} \sim {{{{{\rm{comparison}}}}}}\,{{{{{\rm{of\; interest}}}}}}*{{{{{\rm{variable}}}}}}\,{{{{{\rm{to}}}}}}\,{{{{{\rm{control}}}}}}+(1/{{{{{\rm{individual}}}}}})$$

This model robustly tests for differential gene expression for comparisons of interest (ex. pSyn- vs. pSyn+, brain region differences, etc.) while controlling for confounding variables and multiple sampling of the same samples.

Differential expression of gene targets in the CosMx experiments was determined via a general effects linear regression model in the MAST framework (10.1186/s13059-015-0844-5). This framework robustly tests for differential gene expression while controlling for variations in cell type detection rates.

#### Gene set enrichment analysis

Gene Set Enrichment Analysis was implemented using the *GSVA*^87^ (RRID:SCR_021058) package in R to determine disrupted pathways between pSyn- and pSyn+ neurons. The minimum pathway size was set to 5 genes and maximum pathway size was set to 500 genes. We utilized Kegg Brite pathways to determine which transcriptional pathways are differentially expressed.

#### Cell-type deconvolution

We used nanoString’s cell-type deconvolution algorithm, *SpatialDecon*^40^, to determine the cell-type proportions in our spatial transcriptomics data. We determined cell-type proportions in our data via comparison of our data to single-cell atlas data from the Allen Brain Institute via *SpatialDecon*’s log normal regression. To determine statistical significance of differences in cell type proportions between inclusion bearing (pSyn+) and non-inclusion bearing (pSyn-) segments, we utilized the following equation:

2 $${{{{{\rm{y}}}}}}={{{{{\rm{cell}}}}}}-{{{{{\rm{type}}}}}}\,{{{{{\rm{proportion}}}}}} \sim {{{{{\rm{inclusion}}}}}}\,{{{{{\rm{status}}}}}}\,{{{{{\rm{group}}}}}}+(1/{{{{{\rm{individual}}}}}})$$

#### Published Protocols

All lab protocols used in this paper are publicly available on protocols.io:

α-Synuclein Protein Preparation (Large scale): 10.17504/protocols.io.bp2l6xwmklqe/v1^88^

Stereotaxic Injections: 10.17504/protocols.io.yxmvm3zy5l3p/v1^89^

Transcardial Perfusion in Mouse: 10.17504/protocols.io.dm6gpbrw1lzp/v1^90^

Immunohistochemistry/Immunofluorescence: 10.17504/protocols.io.5jyl89m9dv2w/v1^91^

Dual In Situ Hybridization/Immunofluorescence: 10.17504/protocols.io.bp2l61n91vqe/v1^92^

QUINT Workflow for Fluorescence: 10.17504/protocols.io.4r3l22y6jl1y/v1^93^

### Reporting summary

Further information on research design is available in the Nature Portfolio Reporting Summary linked to this article.

### Supplementary information

Supplementary Information

Peer Review File

Reporting Summary

### Source data

Source data

## Data Availability

The raw sequencing files and associated metadata for both mouse and human experiments generated in this study have been deposited in the Sequence Read Archive (SRA): https://www.ncbi.nlm.nih.gov/sra/PRJNA1082339. The cell type and pathology data generated in this study are provided in the Source Data file. The pre-quality control and supporting data for all experiments are available at the following Zenodo repository: https://zenodo.org/records/10729767^94^ Source data are provided with this paper.

## Code availability

Code used to generate the analyze gene expression and cell detection is available on GitHub: https://github.com/Goralsth/Spatial-transcriptomics-reveals-molecular-dysfunction-associated-with-cortical-Lewy-pathology^95^ (10.5281/zenodo.10732492), https://github.com/DaniellaDeWeerd/NutilToUsable^96^ (10.5281/zenodo.10626455), and https://github.com/DaniellaDeWeerd/ObjectAndRegionSum^97^ (10.5281/zenodo.10650925).
