# Supplementary material for: Spatial transcriptomics reveals molecular dysfunction associated with cortical Lewy pathology
Source: Nat Commun. 2024 Mar 26;15:2642. doi: 10.1038/s41467-024-47027-8 (PMC10966039; doi:10.1038/s41467-024-47027-8)
Supplement: Supplementary file 1 — Supplementary Information [file 41467_2024_47027_MOESM1_ESM.pdf]

Supplementary Information For

Spatial transcriptomics reveals molecular dysfunction associated with cortical Lewy pathology

Thomas M. Goralski[1,2], Lindsay Meyerdirk[1,2], Libby Breton[1,2], Laura Brasseur[1], Kevin Kurgat[1,2], Daniella DeWeerd[1,2], Lisa Turner[4], Katelyn Becker[5], Marie Adams[5], Daniel J. Newhouse[6], Michael X. Henderson[1,2]*

[1]Department of Neurodegenerative Science, Van Andel Institute, Grand Rapids, MI 49503
[2]Aligning Science Across Parkinson's (ASAP) Collaborative Research Network, Chevy Chase, MD
[3]Van Andel Institute, Grand Rapids, MI 49503
[4]Van Andel Institute Pathology Core , Grand Rapids, MI 49503
[5]Van Andel Institute Genomics Core, Grand Rapids, MI 49503
[6]NanoString Technologies, Seattle, WA, USA

*Correspondence:
Michael X. Henderson
333 Bostwick Ave NE, Grand Rapids, MI 49503
(616) 234-5489
michael.henderson@vai.org

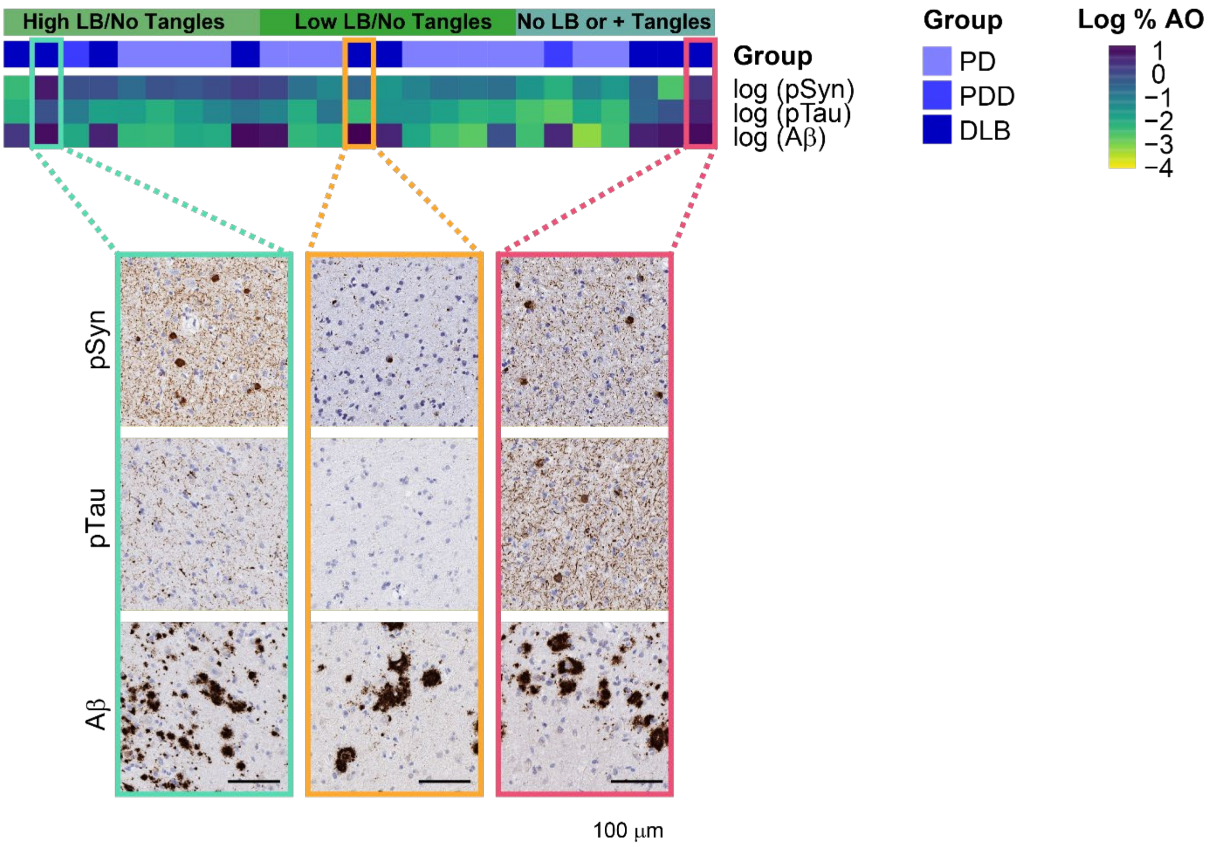

Supplementary Fig. 1. Characterization of human brain tissue Cingulate cortex from 25 PD, PDD, or DLB cases was annotated and the percentage of area occupied with pS129 α-synuclein, pS202/T205 tau (AT8), or Aβ were quantified. Values for this quantitation are shown. pS409/410 TDP-43 was also stained, but no cases showed positive staining. Following quantification, tissue was also manually categorized into one of three categories: 1) high Lewy bodies, no tangles, 2) Low Lewy bodies, no tangles, or 3) no Lewy bodies, or high Lewy bodies, but also with high tau tangles. Examples of each category are below. The left example has high Lewy bodies, and the tau pathology that can be seen is all neuritic. The middle case has no tau pathology, but the Lewy bodies are too sparse to use for spatial transcriptomics. The right case has both high Lewy bodies and high tau tangles, making it unusable for this project. Scale bars = 100 μm. Source data are provided as a Source Data file.

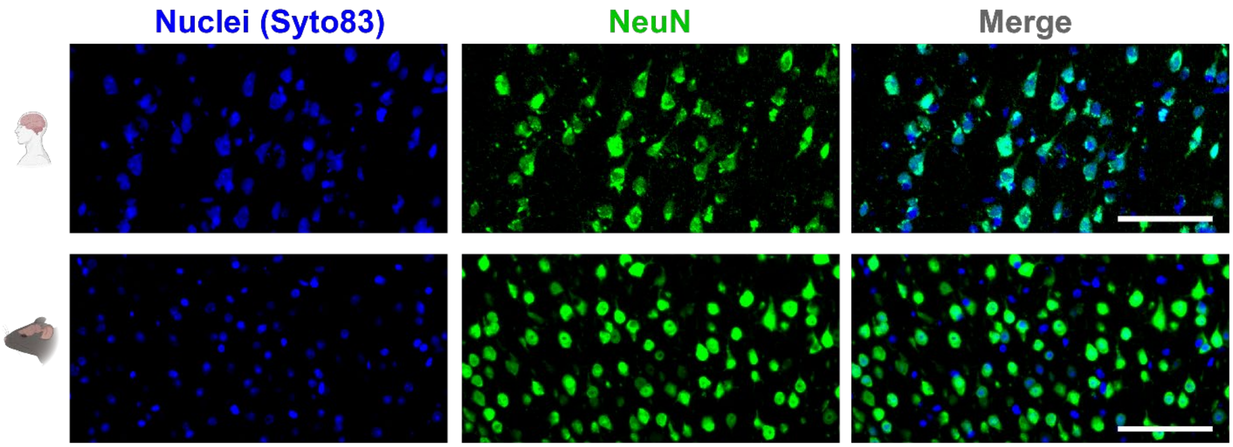

Supplementary Fig. 2. Characterization of NeuN as a morphology marker Both human cingulate cortex and mouse cortex were stained with Syto83 as a nuclear marker and NeuN as a neuronal cell body marker and scanned on the GeoMx instrument. NeuN stains neuronal nuclei, but also expands into the neuronal cytoplasm, so segmentation using this marker would be expected to collect nuclear as well as cytoplasmic RNA transcripts. Created with BioRender.com. Scale bars = 100 µm.

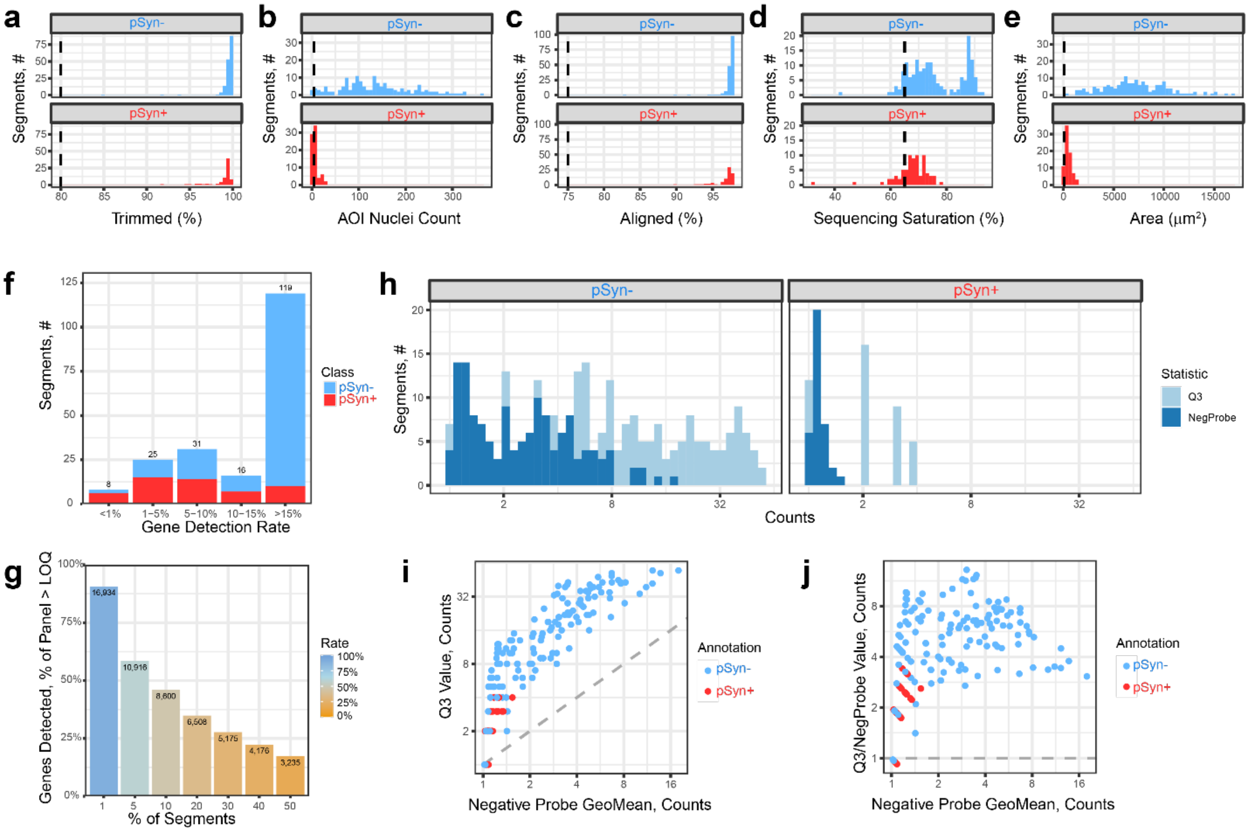

Supplementary Fig. 3. Quality control of human GeoMx data (a-e) Assessment of segments for quality based on spatial information and standard transcriptomics quality control metrics. **(f)** Number of segments with the given percentage of genes detected above the limit of quantification (LOQ). **(g)** Assessment of the number of genes detected above LOQ in the given percentage of segments. **(h)** Assessment of negative probe count values and Q3 values for each segment. **(i)** Comparison of the segments Q3 count value to the geometric mean of the negative probes. **(j)** Comparison of the segments Q3 values divided by their negative probes geometric mean to the negative probe geometric mean.

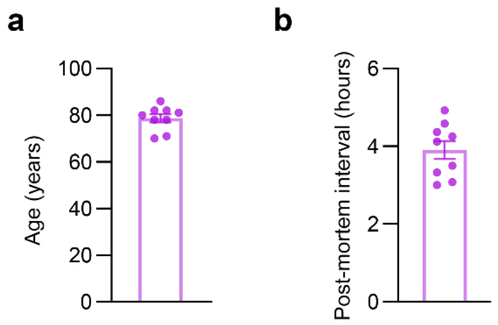

Supplementary Fig. 4 Human brain tissue information (**a**) Age (Mean=78.67, SEM=1.740, n=9) and (**b**) post-mortem interval is shown for PD, PDD, and DLB cases used for GeoMx analysis (Mean= 3.906, SEM 0.2316, n=9). Source data are provided as a Source Data file.

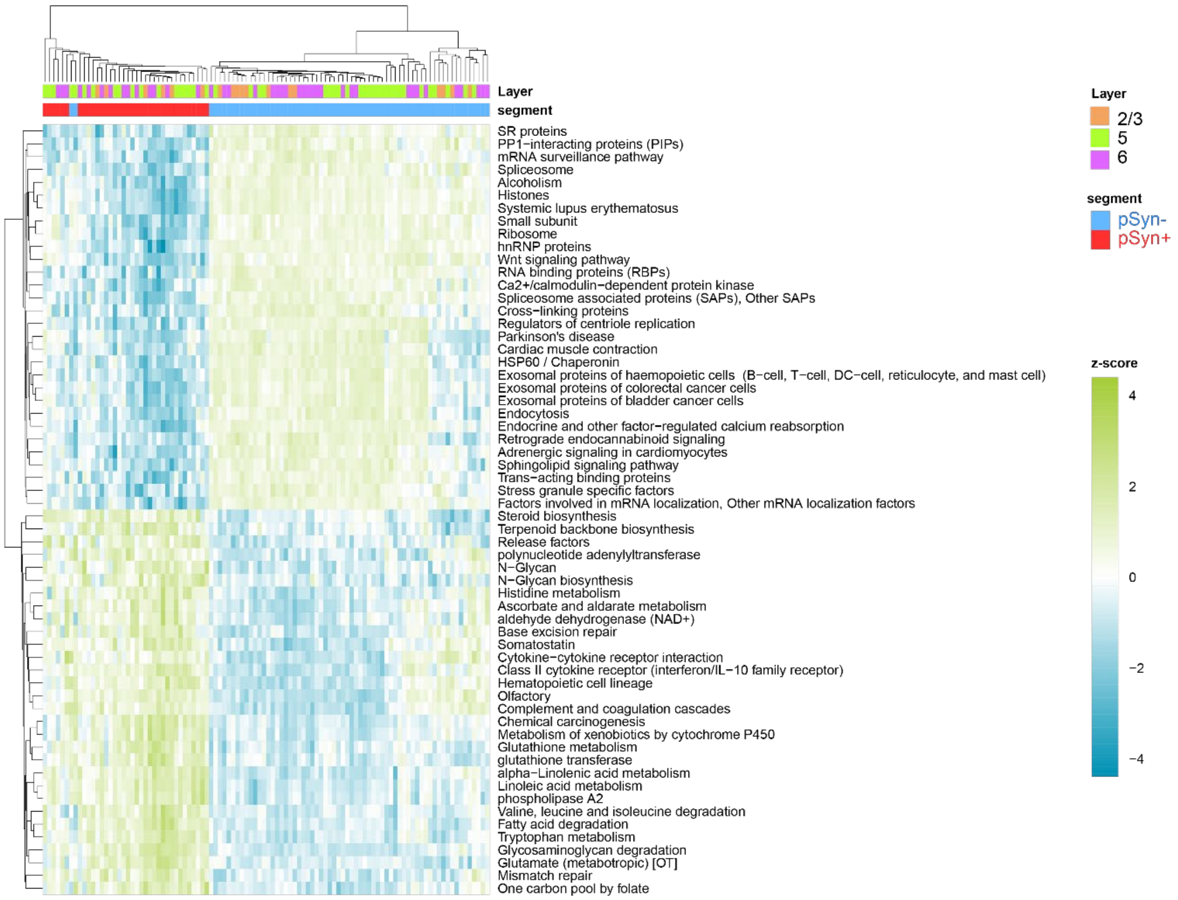

Supplementary Fig. 5 Human gene set enrichment analysis Gene set enrichment analysis was performed on pSyn- and pSyn+ segments in from α-synuclein PFF-injected mice. Z-scores of individual segments are plotted for each pathway. The top 60 pathways enriched in either pSyn- or pSyn+ segments are plotted.

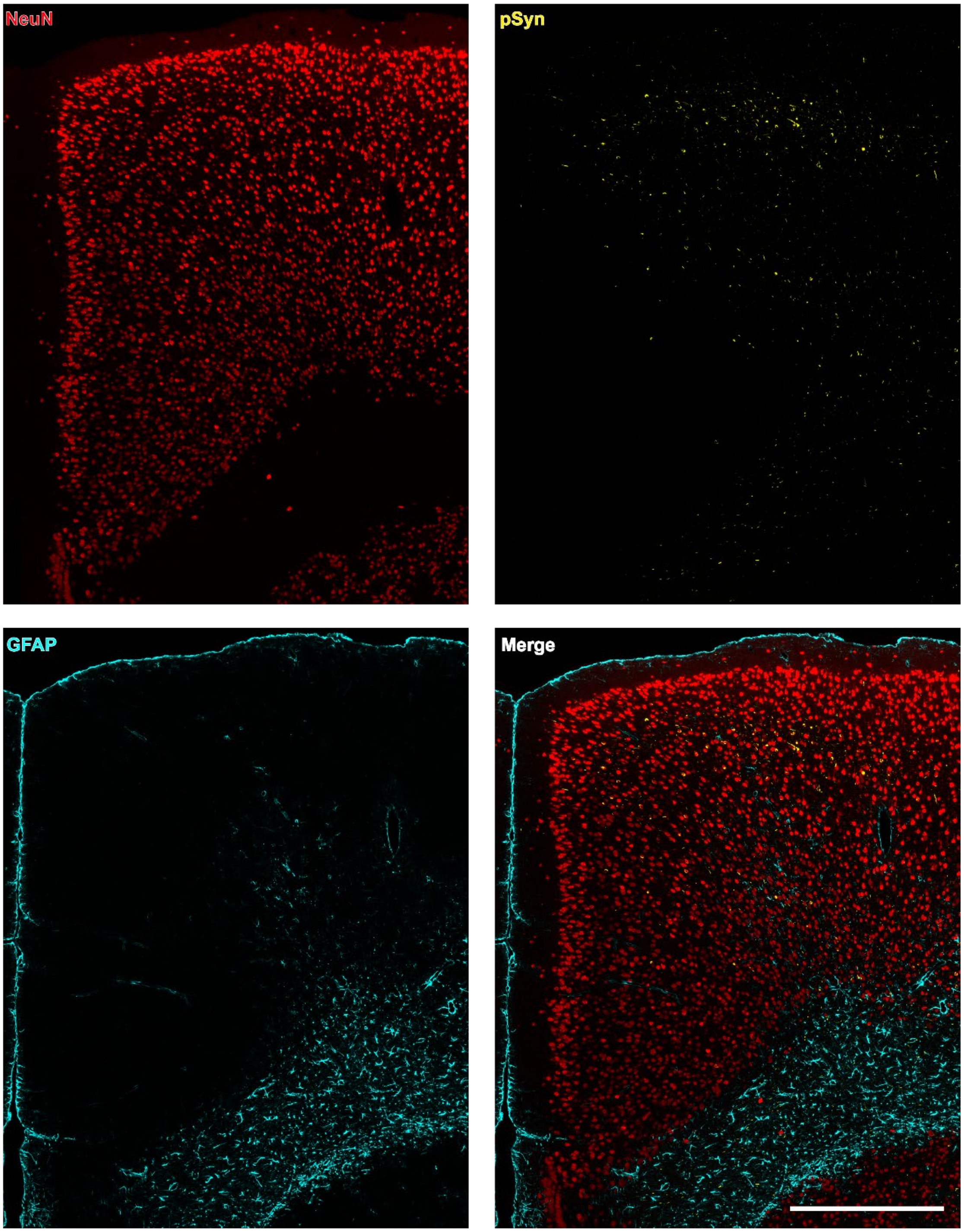

Supplementary Fig. 6. Representative cortex from α-synuclein PFF-injected mouse Three different proteins were stained for segmentation on the GeoMx DSP instrument. NeuN stains neuron cell bodies. pSyn stains α-synuclein pathology. GFAP stains for astrocytes. pSyn pathology is primarily observed in upper layer 5 and in layer 6 of cortex. Scale bar = 0.5 mm.

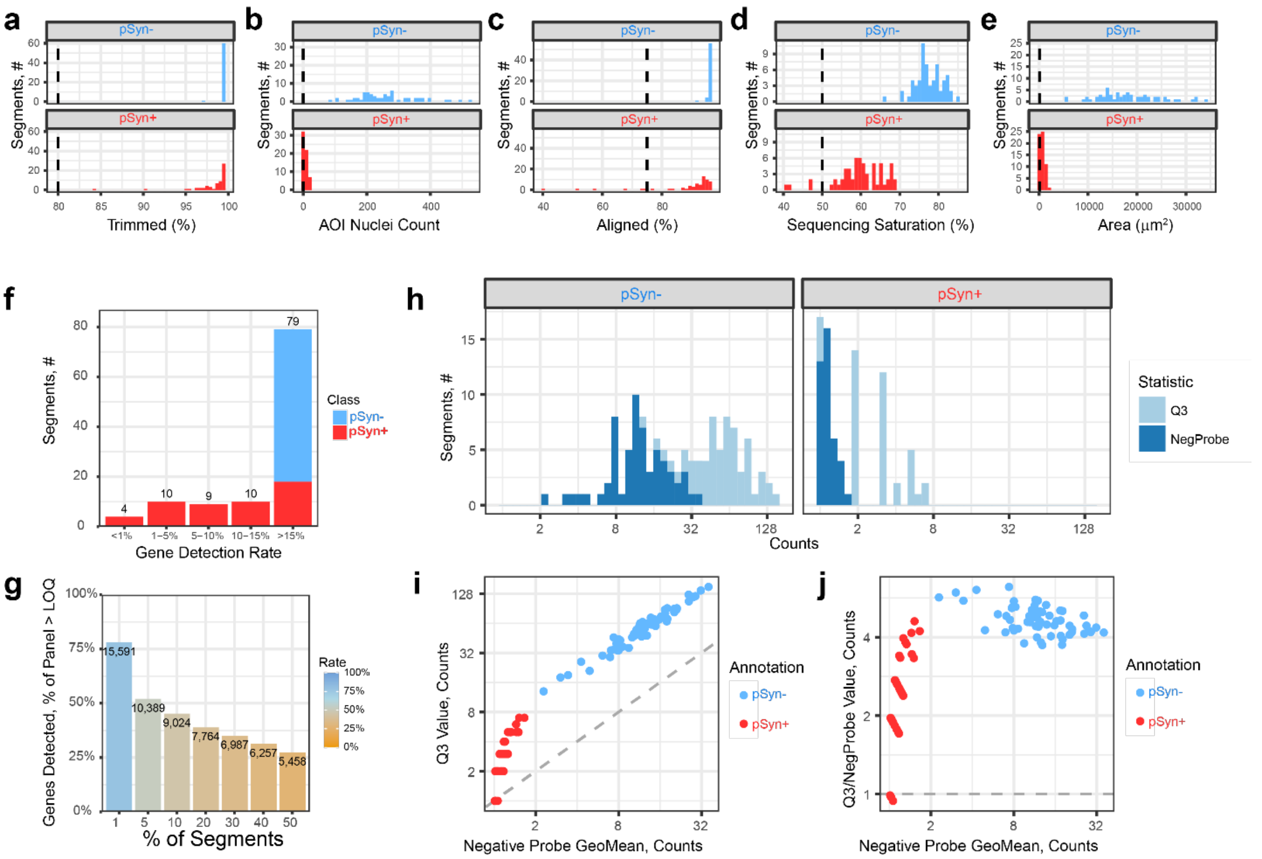

Supplementary Fig. 7. Quality control of mouse GeoMx data (a-e) Assessment of segments for quality based on spatial information and standard transcriptomics quality control metrics. **(f)** Number of segments with the given percentage of genes detected above the limit of quantification (LOQ). **(g)** Assessment of the number of genes detected above LOQ in the given percentage of segments. **(h)** Assessment of negative probe count values and Q3 values for each segment. **(i)** Comparison of the segments Q3 count value to the geometric mean of the negative probes. **(j)** Comparison of the segments Q3 values divided by their negative probes geometric mean to the negative probe geometric mean.

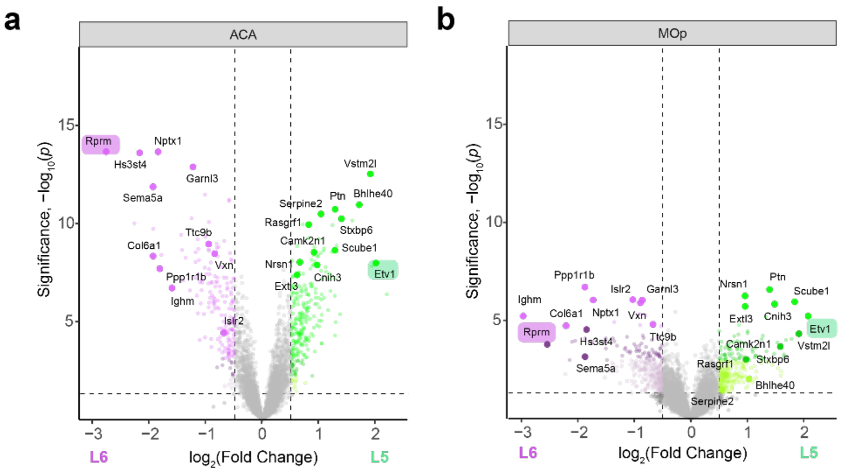

Supplementary Fig. 8. Differential gene expression by layer and cell types (**a**) Volcano plot comparing genes differentially expressed between NeuN segments in layer 5 and layer 6 of ACA identifies genes know to be differentially expressed in the different cortical layers. Two such genes are highlighted. (**b**) Volcano plot comparing genes differentially expressed between NeuN segments in layer 5 and layer 6 of MOp identifies genes know to be differentially expressed in the different cortical layers. Two such genes are highlighted.

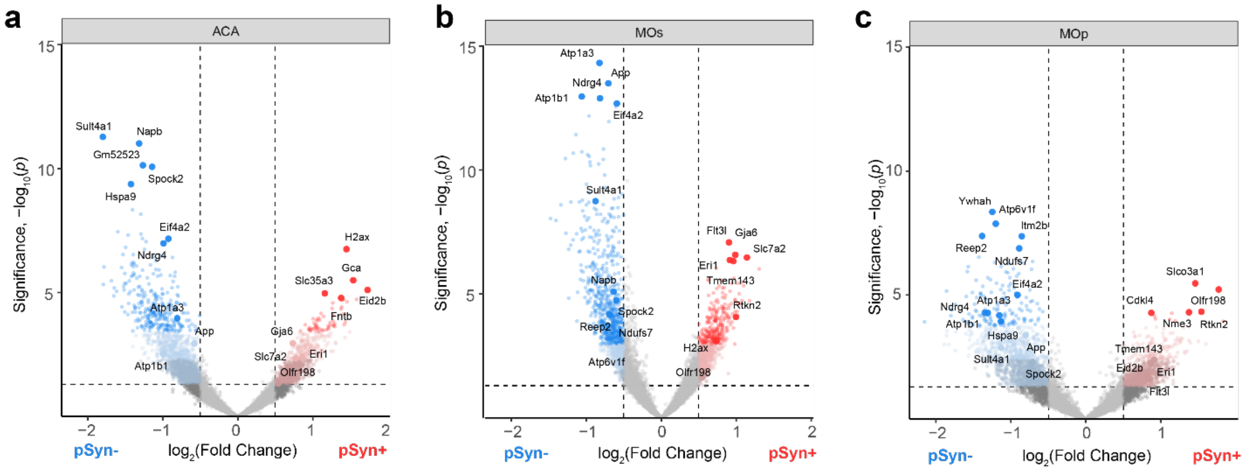

Supplementary Fig. 9. α-Synuclein inclusion-bearing neurons show conserved gene expression changes associated with cellular dysfunction in three cortical regions Volcano plot comparing genes differentially expressed between pSyn- and pSyn+ segments in the ACA (**a**), MOs (**b**), or MOp (**c**) regions with the top 30 DEGs labeled.

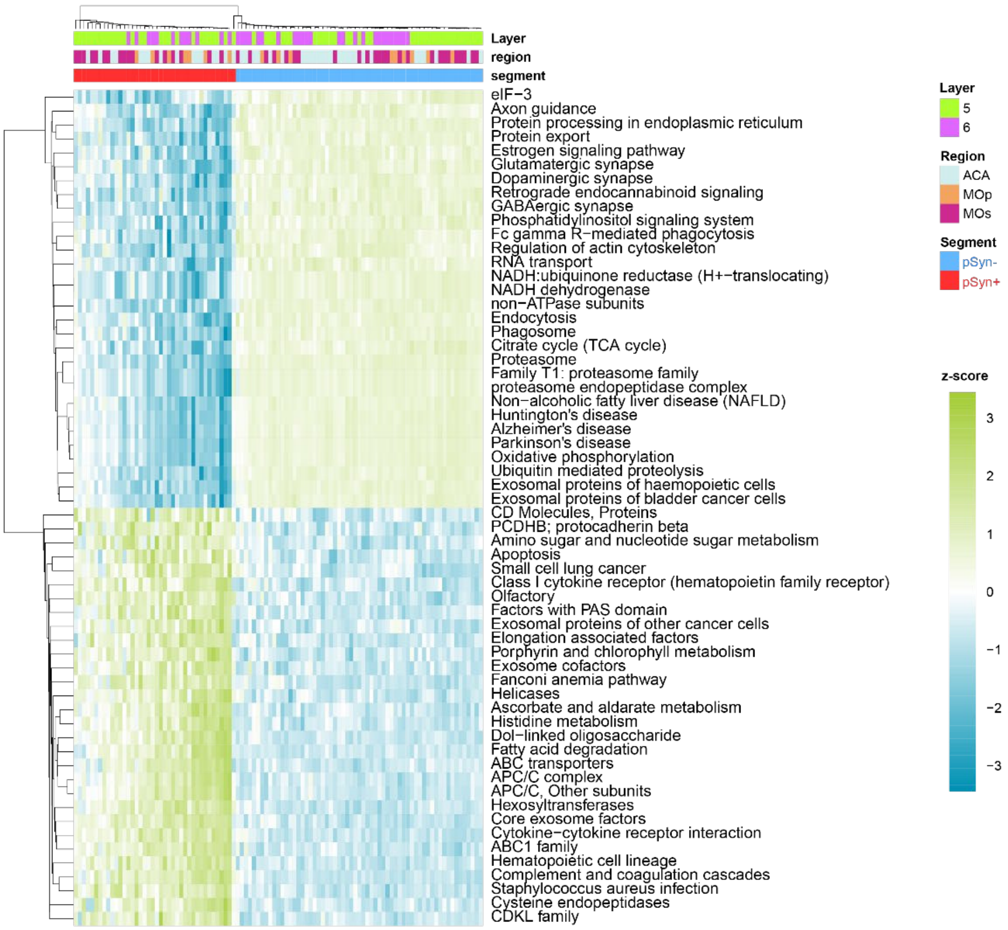

Supplementary Fig. 10. Mouse gene set enrichment analysis Gene set enrichment analysis was performed on NeuN and pSyn segments from α-synuclein PFF-injected mice. Z-scores of individual segments are plotted for each pathway. The top 60 pathways enriched in either pSyn or NeuN segments are plotted.

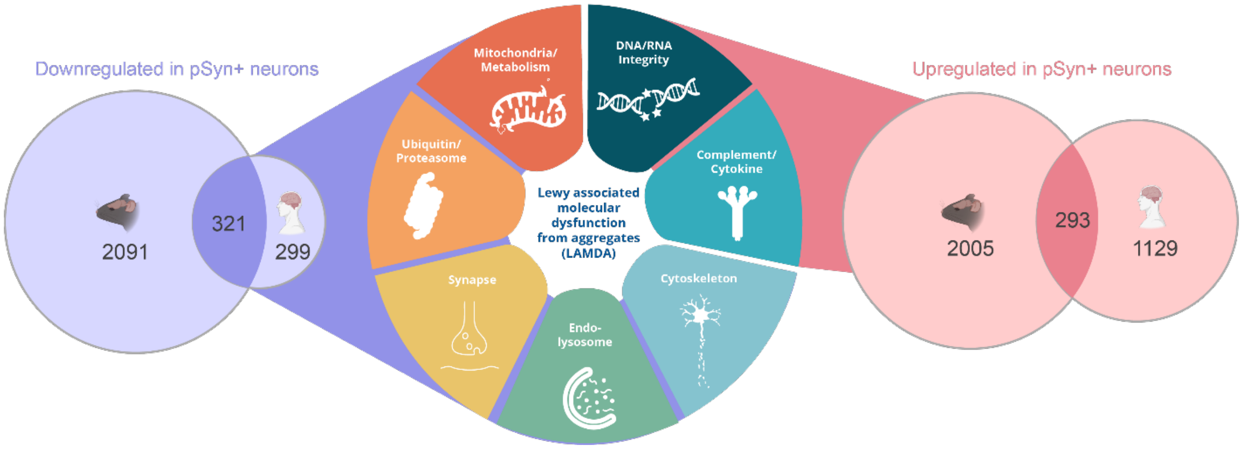

Supplementary Fig. 11. Lewy-associated molecular dysfunction from aggregates (LAMDA) Numbers of genes downregulated or upregulated in pSyn inclusion-bearing neurons are plotted from mouse and human tissue. The overlapping area is representative of the genes which show conserved expression changes in mice and humans. Conserved upregulated and downregulated genes in mouse and human fall with certain pathways and are described as a Lewy associated molecular dysfunction from aggregates (LAMDA) signature. Source data are provided as a Source Data file. Created with BioRender.com.

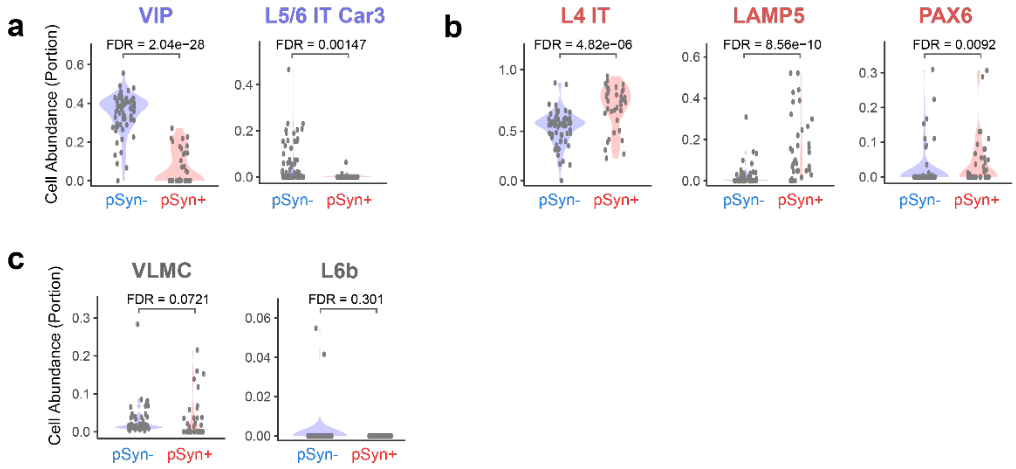

Supplementary Fig. 12. α-Synuclein pathology is enriched in IT neurons (**a**) Cell abundance estimates from cell deconvolution analysis from human cingulate cortex shows enrichment of VIP and L5/6 Car3 neurons in pSy- neurons. (**b**) Cell abundance estimates from cell deconvolution analysis from human cingulate cortex shows enrichment of L4 IT, LAMP5, and PAX6 neurons in pSyn+ neurons. (**c**) VLMC and L6b neurons were identified at lower abundances and not found to be enriched in either group.

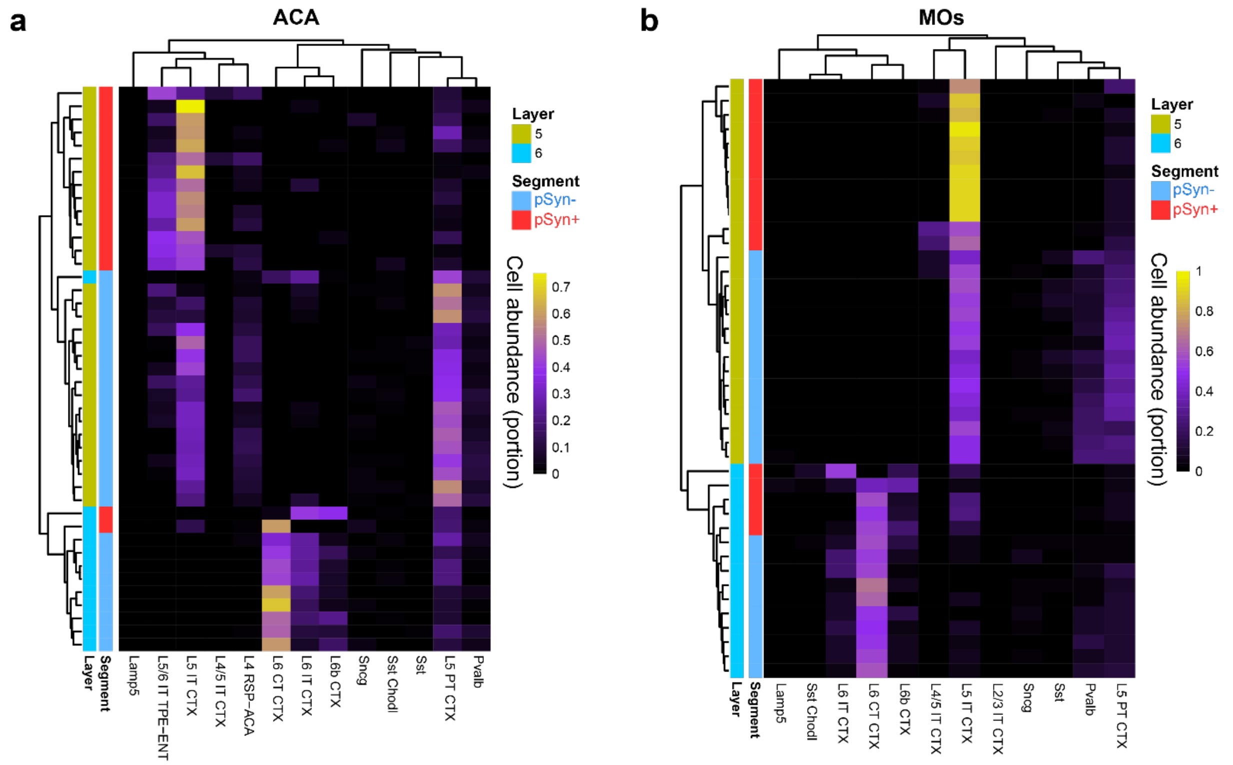

Supplementary Fig. 13. Cell deconvolution plots (**a**) The relative abundance of cell types was calculated via cell deconvolution for each segment in the ACA region. Layer 5 and layer 6 segments largely cluster separately, with pSyn- and pSyn+ segments clustering within their respective layers. Layer 5 pSyn+ segments are largely L5 IT or L5/6 IT TPE-ENT cell types, while layer 5 pSyn- segments show less abundance of L5 IT and a higher proportion of L5 PT neurons. Layer 6 pSyn+ segments are a mix of L6 CT and L6b neurons, while layer 6 pSyn- segments are mostly L6 CT and L6 IT neurons. Similar results are seen for the MOs region (**b**).

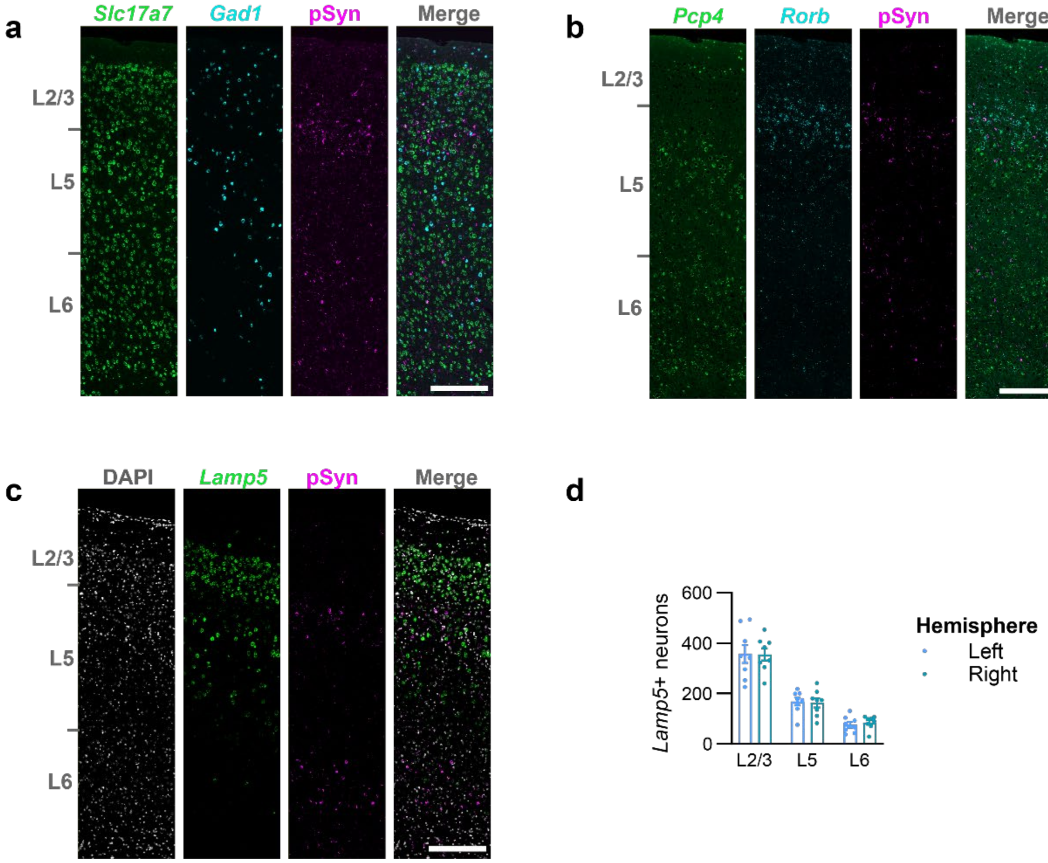

Supplementary Fig. 14. α-Synuclein pathology is enriched in layer 5 intratelencephalic and layer 6b neurons (**a**) A whole slice through mouse cortex (primarily MOs) stained for *Slc17a7*, *Gad1*, and pSyn. Scale bar = 250 µm. (**b**) A whole slice through mouse cortex (primarily MOs) stained for *Pcp4*, *Rorb*, and pSyn. Scale bar = 250 µm. (**c**) A whole slice through mouse cortex (primarily MOs) stained for DAPI, *Lamp5*, and pSyn. Scale bars = 250 µm. (**d**) To determine if lower *Lamp5* expression in inclusion-bearing neurons was related to cell type differences or phenotypic downregulation of *Lamp5*, the number of low *Lamp5*-expressing cells was quantified on the left and right hemisphere of mice. No difference was detected between the number of low *Lamp5*-expressing cells between hemispheres. Two-way ANOVA showed a significant effect of cortical layers ($p<0.0001$), but not between hemispheres ($p=0.9595$) with Sidak's multiple comparisons test showing no effect of hemisphere within cortical layers (L2/3: $p>0.9999$, L5: $p=0.9975$, L6: $p=0.9867$) (L2/3 Left Mean=356.250, SEM=35.525,n=8.; L2/3 Left Mean=355, SEM=24.126, n=8; L5 Right Mean=167.875, SEM=15.877,n=8; L5 Left Mean=162.750, SEM=18.204, n=8; L6 Right Mean=75.750, SEM=11.331,n=8; L6 Left Mean=84.75, SEM=9.344,n=8). Source data are provided as a Source Data file.

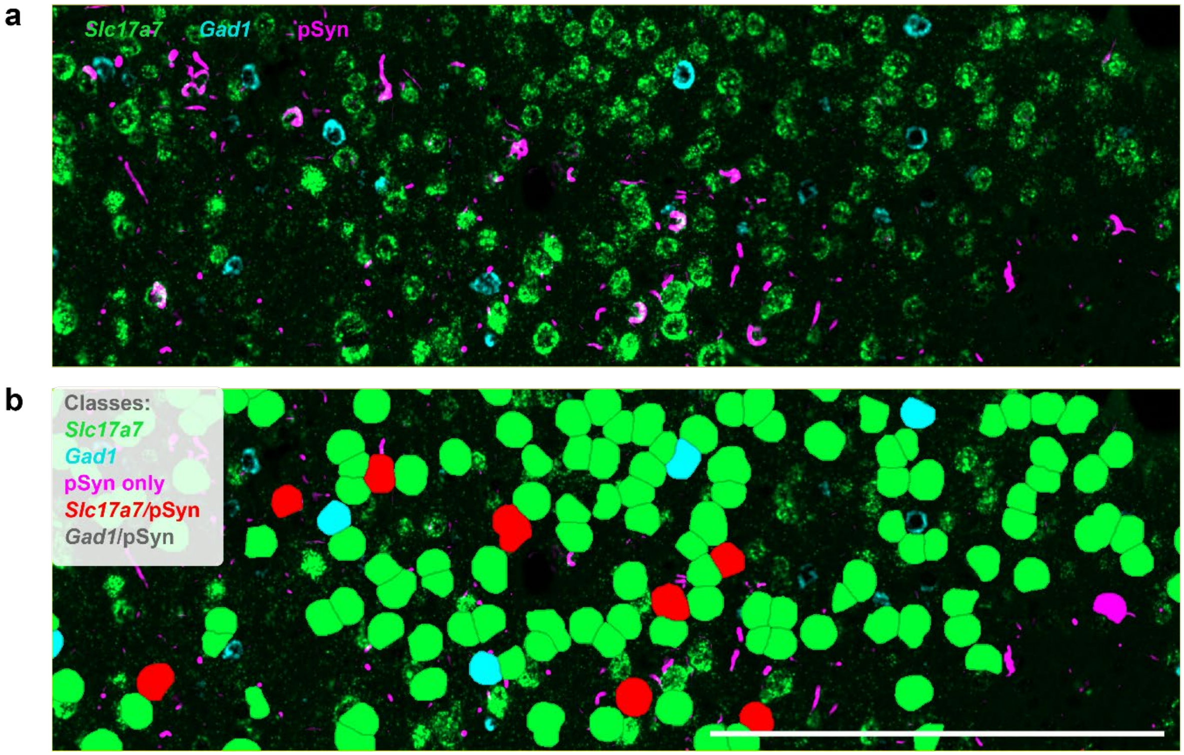

Supplementary Fig. 15 Cell classification in mouse cortex (**a**) Mouse cortex stained for RNAs *Slc17a7* and *Gad1* and protein pSyn. (**b**) Cells from panel **a**, identified by nuclear DAPI signal and classified based on the presence or absence of *Slc17a7*, *Gad1*, or pSyn in the cytoplasm. Cells with more than one marker were classified as having multiple markers. The overlay indicates the cell class. Scale bar = 250 µm.

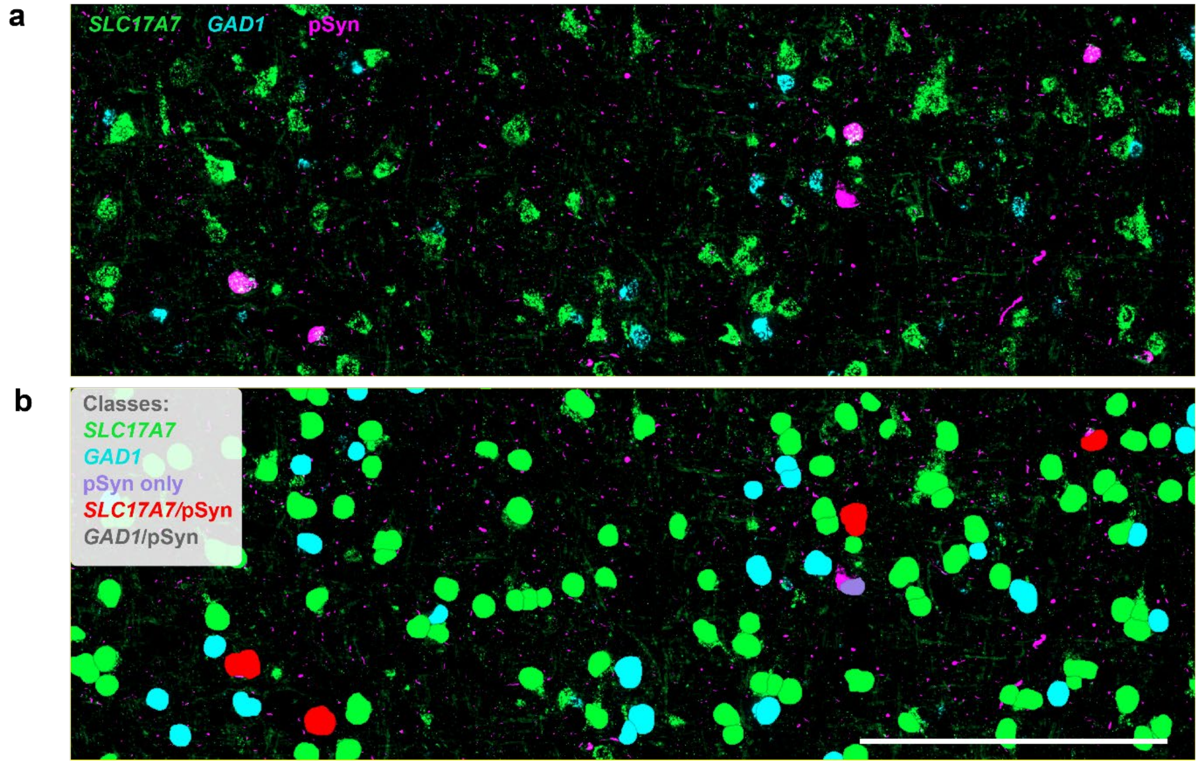

Supplementary Fig. 16 Cell classification in human cortex (**a**) Human cingulate cortex stained for *SLC17A7* and *GAD1* RNA transcripts and protein pSyn. (**b**) Cells from panel **a**, identified by nuclear DAPI signal and classified based on the presence or absence of *SLC17A7*, *GAD1*, or pSyn in the cytoplasm. Cells with more than one marker were classified as having multiple markers. The overlay indicates the cell class. Scale bar = 250 µm.

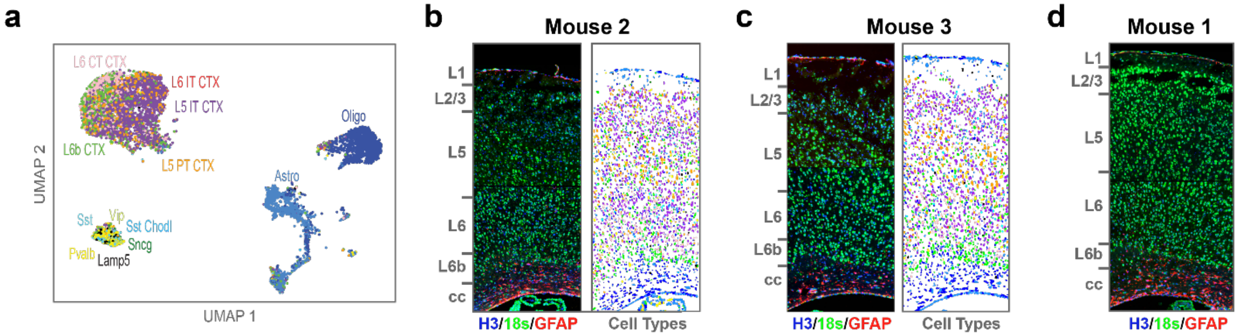

Supplementary Fig. 17. Single cell spatial transcriptomics of pathological mouse cortex (**a**) UMAP dimension reduction plot showing the clustering of distinct cell types based on high-dimensional transcriptomics data from 3 mouse cortex samples. (**b**, **c**) Data from mouse 2 and 3 showing the morphology markers that were used for cell segmentation (Histone H3, 18s rRNA, GFAP) and the colored cell overlays noting cell type. The color of cell types is the same as the UMAP in panel **a**. (**d**) Data from mouse 3 showing the morphology markers as in panels **b** and **c**. The cell type overlay for mouse 1 is displayed in **Fig. 8b**.

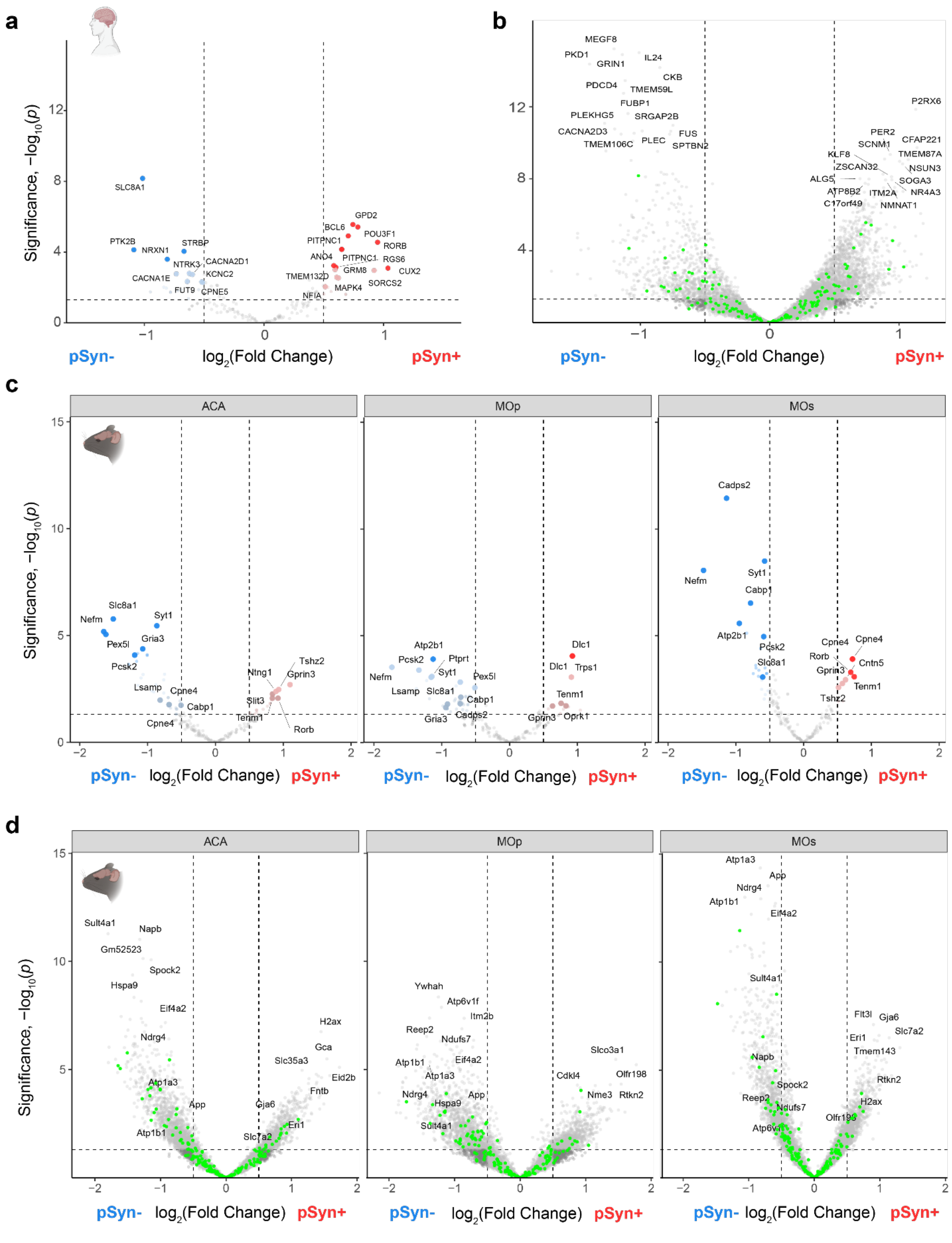

Supplementary Fig. 18 Differential expression of cell type genes (**a**) Volcano plot comparing genes differentially expressed between pSyn- and pSyn+ segments in the human cingulate cortex, but only

comparing genes known to be differentially expressed by different cell types. (**b**) Volcano plot comparing all genes differentially expressed between pSyn- and pSyn+ segments in the human cingulate cortex with cell type selective genes highlighted in green. Note that cell type genes are a small percentage (1.5%) of all DEGs. (**c**) Volcano plots comparing genes differentially expressed between pSyn- and pSyn+ segments in three regions of mouse cortex, but only comparing genes known to be differentially expressed by different cell types. (**d**) Volcano plot comparing all genes differentially expressed between pSyn- and pSyn+ segments in three regions of mouse cortex with cell type selective genes highlighted in green. Note that cell type genes are a small percentage (1.0%) of all DEGs. Created with BioRender.com.
