# Supplementary material for: Spatial transcriptomics reveals molecular dysfunction associated with cortical Lewy pathology
Source: Nat Commun. 2024 Mar 26;15:2642. doi: 10.1038/s41467-024-47027-8 (PMC10966039; doi:10.1038/s41467-024-47027-8)
Supplement: Supplementary file 3 — Reporting Summary [file 41467_2024_47027_MOESM3_ESM.pdf]

nature portfolio

Corresponding author(s): Michael X. Henderson

Last updated by author(s): Mar 4, 2024

Reporting Summary

Nature Portfolio wishes to improve the reproducibility of the work that we publish. This form provides structure for consistency and transparency in reporting. For further information on Nature Portfolio policies, see our Editorial Policies and the Editorial Policy Checklist.

Statistics

For all statistical analyses, confirm that the following items are present in the figure legend, table legend, main text, or Methods section.

n/a	Confirmed	
☐	☒	The exact sample size (*n*) for each experimental group/condition, given as a discrete number and unit of measurement
☐	☒	A statement on whether measurements were taken from distinct samples or whether the same sample was measured repeatedly
☐	☒	The statistical test(s) used AND whether they are one- or two-sided<br>*Only common tests should be described solely by name; describe more complex techniques in the Methods section.*
☐	☒	A description of all covariates tested
☐	☒	A description of any assumptions or corrections, such as tests of normality and adjustment for multiple comparisons
☐	☒	A full description of the statistical parameters including central tendency (e.g. means) or other basic estimates (e.g. regression coefficient) AND variation (e.g. standard deviation) or associated estimates of uncertainty (e.g. confidence intervals)
☐	☒	For null hypothesis testing, the test statistic (e.g. *F*, *t*, *r*) with confidence intervals, effect sizes, degrees of freedom and *P* value noted<br>*Give P values as exact values whenever suitable.*
☒	☐	For Bayesian analysis, information on the choice of priors and Markov chain Monte Carlo settings
☒	☐	For hierarchical and complex designs, identification of the appropriate level for tests and full reporting of outcomes
☒	☐	Estimates of effect sizes (e.g. Cohen's *d*, Pearson's *r*), indicating how they were calculated

Our web collection on statistics for biologists contains articles on many of the points above.

Software and code

Policy information about availability of computer code

Data collection	All GeoMx data was collected from the GeoMx digital spatial profiler (DSP).
Data analysis	All data was analyzed in either GraphPad Prism 9 or R software. The packages and code used for analysis are available at the following locations: analyze gene expression and cell detection is available on GitHub: https://github.com/Goralsth/Spatial-transcriptomics-reveals-molecular-dysfunction-associated-with-cortical-Lewy-pathology (DOI: 10.5281/zenodo.10732492) , https://github.com/DaniellaDeWeerd/NutilToUsable (DOI: 10.5281/zenodo.10626455), and https://github.com/DaniellaDeWeerd/ObjectAndRegionSum (DOI: 10.5281/zenodo.10650925).

For manuscripts utilizing custom algorithms or software that are central to the research but not yet described in published literature, software must be made available to editors and reviewers. We strongly encourage code deposition in a community repository (e.g. GitHub). See the Nature Portfolio guidelines for submitting code & software for further information.

Data

Policy information about availability of data

All manuscripts must include a data availability statement. This statement should provide the following information, where applicable:

- Accession codes, unique identifiers, or web links for publicly available datasets
- A description of any restrictions on data availability
- For clinical datasets or third party data, please ensure that the statement adheres to our policy

The raw sequencing files and associated metadata for both mouse and human experiments are available at the following Sequence Read Archive (SRA) webpage:

https://www.ncbi.nlm.nih.gov/sra/PRJNA1082339
The pre-quality control and supporting data for all experiments are available at the following Zenodo repository: https://zenodo.org/records/10729767 (DOI: 10.5281/zenodo.10729767).

Research involving human participants, their data, or biological material

Policy information about studies with human participants or human data. See also policy information about sex, gender (identity/presentation), and sexual orientation and race, ethnicity and racism.

Reporting on sex and gender	No human participants were involved in this study. Human brain tissue was used from both males and females.
Reporting on race, ethnicity, or other socially relevant groupings	No human participants were involved in this study. Human brain tissue race and ethnicity are not reported.
Population characteristics	No human participants were involved in this study.
Recruitment	No human participants were involved in this study.
Ethics oversight	No human participants were involved in this study. All procedures were done in accordance with local institutional review board guidelines of the Banner Sun Health Research Institute.

Note that full information on the approval of the study protocol must also be provided in the manuscript.

Field-specific reporting

Please select the one below that is the best fit for your research. If you are not sure, read the appropriate sections before making your selection.

[x] Life sciences [ ] Behavioural & social sciences [ ] Ecological, evolutionary & environmental sciences

For a reference copy of the document with all sections, see nature.com/documents/nr-reporting-summary-flat.pdf

Life sciences study design

All studies must disclose on these points even when the disclosure is negative.

Sample size	Sample size was determined based on pilot data. Significant results were found with as few as 3 samples, but based on manufacturer recommendations, we increased the number of samples to ensure the reproducibility of results.
Data exclusions	Data were excluded that did not meet the defined quality control metrics as outlined in the manuscript.
Replication	Replication of differential gene expression was considered across cortical layers and across cortical regions. The final analyses all involved gene expression patterns that were conserved across cortical layer and brain region. No independent replication in a separate cohort of mice or human cases was undertaken.
Randomization	All studies are in within-subject studies. That is, comparisons are made for two different cell types within the same subjects.
Blinding	For cell type analysis, individuals were blinded to the expected outcomes. No separate groups were included since all cell types were from the same cases, so no blinding was possible.

Reporting for specific materials, systems and methods

We require information from authors about some types of materials, experimental systems and methods used in many studies. Here, indicate whether each material, system or method listed is relevant to your study. If you are not sure if a list item applies to your research, read the appropriate section before selecting a response.

Materials & experimental systems

n/a	Involved in the study	
[ ]	[x]	Antibodies
[x]	[ ]	Eukaryotic cell lines
[x]	[ ]	Palaeontology and archaeology
[ ]	[x]	Animals and other organisms
[x]	[ ]	Clinical data
[x]	[ ]	Dual use research of concern
[x]	[ ]	Plants

Methods

n/a	Involved in the study	
[x]	[ ]	ChIP-seq
[x]	[ ]	Flow cytometry
[x]	[ ]	MRI-based neuroimaging

Antibodies

Antibodies used	(Primary antibodies: SMI-32, Biolegend 801702, 1:250, SATB2, Abcam ab92446 1:250, pS129 α-synuclein (81A, Biolegend 825701, 1:1000). Secondary antibodies: Secondaries: GαM IgG1 488 (Invitrogen A21121, RRID:AB_2535764, 1:1000), GαRb 546 (Invitrogen A11010, RRID:AB_2534077, 1:1000), GαIgG2a 647 (Invitrogen A21241, RRID:AB_2535810, 1:1000), GαM IgG2a (Thermo Scientific A21241, RRID:AB_2535810, 1:400).
Validation	Antibodies were all reported as applicable for immunohistochemistry in mouse and human tissue. In addition, antibodies were validated in-house by a series of experiments using different concentrations of antibodies prior to their use in designed experiments.

Animals and other research organisms

Policy information about studies involving animals; ARRIVE guidelines recommended for reporting animal research, and Sex and Gender in Research

Laboratory animals	C57BL/6J Mus musculus (Jackson Laboratory (000664; RRID:IMSR_JAX:000664)) injected at 3 months of age and aged a further 3 months
Wild animals	No wild animals were used in this study.
Reporting on sex	Mice were balanced by sex
Field-collected samples	No field-collected samples were used in this study.
Ethics oversight	All housing, breeding, and procedures were performed according to the NIH Guide for the Care and Use of Experimental Animals and approved by the Van Andel Institute Institutional Animal Care and Use Committee (IACUC).

Note that full information on the approval of the study protocol must also be provided in the manuscript.
